#  Proteomic profiling reveals that DPP4 overexpression increases cell adhesion, inhibits cell migration, and restores androgen sensitivity in prostate cancer

**DOI:** 10.1042/BSR20260054

**Published:** 2026-09-04

**Authors:** José María Mora-Rodríguez, Belén G. Sánchez, Alicia Bort, Alba Díaz-Yuste, Alicia Milla, Julie Courraud, Jerome Zoidakis, Inés Díaz-Laviada

**Affiliations:** 1 University of Alcalá, School of Medicine and Health Sciences, Department of Systems Biology, Biochemistry and Molecular Biology Unit, 28871 Alcalá de Henares, Madrid, Spain; 2 Health Research Institute of Castilla-La Mancha (IDISCAM), Toledo, Castilla-La Mancha, Spain; 3 Institute of Food Science Research, CIAL (CSIC-UAM), Madrid, Spain; 4 Section of Clinical Therapeutics, Department of Medicine, National and Kapodistrian University of Athens, Alexandra Hospital, Athens, Greece; 5 Proteomics Core Facility, School of Science, National and Kapodistrian University of Athens, Athens, Greece; 6 Section of Biochemistry and Molecular Biology, Department of Biology, National and Kapodistrian University of Athens, Athens, Greece; 7 Proteomics Laboratory, Biomedical Research Foundation, Academy of Athens, Athens, Greece

**Keywords:** androgen resistance, capsaicin, CD26, Dipeptidyl peptidase 4, prostate cancer

## Abstract

Dipeptidyl peptidase-4 (DPP4), a serine protease with both enzymatic and non-enzymatic roles, has emerged as a context-dependent modulator of tumor progression. In the present study, we investigated the expression and function of DPP4 in androgen-sensitive and castration-resistant prostate cancer (CRPC) models. Proteomic analysis of androgen-resistant prostate cells overexpressing DPP4 identified the involvement of the cellular adhesion molecules pathway. In prostate cells, lentiviral-mediated DPP4 overexpression restored androgen receptor signaling, inhibited epithelial-to-mesenchymal transition, and reduced cell migration, whereas DPP4 silencing produced the opposite effects. We demonstrate that DPP4 expression is down-regulated in CRPC cells and that treatment with capsaicin (CAP), a bioactive compound derived from red peppers, restores DPP4 expression. Moreover, DPP4 restoration by CAP suppresses prostate tumorigenesis in the TRAMP mice *in vivo* model of prostate cancer. Our results suggest that DPP4 could be a new target for CRPC.

## Introduction

Prostate cancer (PC) is the second most commonly diagnosed malignancy in men worldwide and represents a major cause of cancer-related mortality, particularly in Western countries [1,2]. Despite significant advancements in early detection and treatment, men invariably progress to castration-resistant prostate cancer (CRPC), and AR reactivation contributes to progression in most cases. Androgen deprivation therapy (ADT) remains the cornerstone treatment for both locally advanced and metastatic PC. While ADT initially induces tumor regression or stabilization, the majority of patients ultimately develop a lethal castration-resistant state [3].

Dipeptidyl peptidase 4 (DPP4) is a serine protease widely expressed in most cell types that is involved in multiple physiological functions. In T lymphocytes, DPP4 serves as a marker of activation and is also known as the T-cell activation antigen CD26 or adenosine deaminase binding protein, reflecting its multifunctional nature. DPP4 enzymatically degrades a variety of polypeptides, preferentially when containing a proline or alanine residue at the penultimate position of the amino-terminal region, thereby releasing a dipeptide [4]. These substrates include chemokines, growth factors, fibronectin, neuropeptides, and incretins such as glucagon-like peptide-1 and glucose-dependent insulinotropic peptide [5], which have led to the development of DPP4 inhibitors as antidiabetic agents.

In addition to its enzymatic function, DPP4 modulates cellular processes through non-enzymatic interactions with proteins such as adenosine deaminase, extracellular matrix components, and caveolin-1, triggering downstream effects that modulate the immune response, cell adhesion, and chemokine activity. By interacting with these proteins, DPP4 may modulate different cellular processes independently of its enzymatic activity [6].

However, the role of DPP4 in cancer remains controversial, as it has been implicated in both tumor suppression and tumor promotion [7]. Epidemiological studies have reported that DPP4 inhibitors, known as gliptins, are associated with a 72% reduction in PC risk in patients with type 2 diabetes [8,9] and may confer a significant survival advantage [10]. Despite evidence suggesting a protective role of DPP4 inhibitors, decreased DPP4 expression has been associated with progression to castration resistance. For example, Russo et al. demonstrated that DPP4 expression is significantly down-regulated during the transition to CRPC in a mouse xenotransplantation model of human PC (VCaP cells). This reduction was reversible upon testosterone administration, suggesting that epigenetic mechanisms are involved in its regulation [11]. The study identified DPP4 as an AR-stimulated tumor suppressor gene whose expression declines with progression to castration-resistant PC. Likewise, the down-regulation of DPP4 accelerates the progression to CRPC. Consistently, lower DPP4 expression correlates with higher Gleason scores, advanced pathological stage, tumor metastasis, and shorter progression-free survival in PC patients [12,13]. Furthermore, DPP4 overexpression suppresses the migration and invasion of metastatic PC3 PC cells [14,15]. Reduced DPP4 activity has also been observed in the sera of patients with metastatic PC compared with those with localized disease or healthy controls, as well as in aggressive PC animal models relative to cancer-free counterparts [13]. These findings support the role of DPP4 as a tumor suppressor in castration-resistant PC. Additionally, DPP4 expression has been linked to prostate-specific antigen (PSA) levels and cancer stage, with some studies suggesting that it serves as a negative prognostic indicator for PC [16]. Therefore, it seems that DPP4 exerts context-dependent effects in tumor cells, whereby its proteolytic activity mediates signaling pathways distinct from those regulated by its chaperone function, with the protein interaction activity becoming dominant in the androgen-resistant stage. However, given the conflicting evidence regarding the effects of DPP4 inhibition versus its expression in PC progression, further research is needed to elucidate the precise role of DPP4 in PC pathophysiology and its potential as a therapeutic target [17].

Recent studies revealed that natural products hold significant potential as complementary therapies for PC, providing anticancer benefits with minimal risk of side effects. In particular, capsaicin (CAP), the pungent ingredient of hot chili peppers, has demonstrated a beneficial effect against castration-resistant prostate cells [18,19]. Therefore, it would be of interest to investigate whether CAP has any modulatory effect on DPP4 activity or expression in the context of androgen-resistant PC.

In the present study, we investigated the role of DPP4 in the progression of PC from a hormone-sensitive to a castration-resistant state. To this end, we performed a comprehensive set of *in vitro* and *in vivo* experiments, proteomic analyses, and functional studies. Our results reveal that the decrease in DPP4 in androgen-refractory situations may be reversed by CAP and that DPP4 is involved in tumoral prostate cell adhesion.

## Materials and methods

### Materials

CAP and doxycycline (Dox) were obtained from Sigma–Aldrich (St. Louis, MO, U.S.A.). The psPAX2 packaging vector was a gift from Didier Trono (Addgene plasmid #12260; http://n2t.net/addgene:12260; RRID: Addgene_12260). The pCMV-VSV-G envelope vector was a gift from Robert Weinberg (Addgene, plasmid #8454; http://n2t.net/addgene:8454; RRID:Addgene_8454) [20]. The pLEX307-DPP4-puro expression vector was a gift from Alejandro Chavez and Sho Iketani (Addgene plasmid #158451; http://n2t.net/addgene:158451; RRID: Addgene_158451). A custom pLEX307-DPP4-S630A-puro expression vector, encoding the catalytically inactive DPP4 mutant S630A (Ser630Ala), was generated by GenScript (Piscataway, NJ, U.S.A.) by introducing the S630A substitution into the DPP4 coding sequence using oligonucleotide-based site-directed mutagenesis. Custom oligonucleotides were designed to be fully complementary to the target region of the gene in the plasmid, with the exception of the deliberate mismatch at position 630, where the mutation S630A was intended. The final construct was verified by next-generation sequencing against the full reference sequence before delivery. The Dox-inducible Lenti-TetON-AMPK expression plasmid was constructed in-house (see Supplementary Figure S1). The vector harbors the human AMP-activated kinase (AMPK) α1, β1, and γ1 subunit cDNAs within a single backbone, enabling their coordinated and stoichiometric expression upon Dox induction.

### Cell culture

The human PC cell lines LNCaP (ATCC CRL-1740) and PC3 (ATCC CRL-1435) were obtained from the American Type Culture Collection (ATCC, Rockville, MD, U.S.A.). Cells were maintained in RPMI-1640 medium supplemented with 10% fetal bovine serum (FBS) and 1% antibiotic–antimycotic solution (100 IU/ml penicillin G sodium, 100 μg/ml streptomycin sulfate, and 0.25 μg/ml amphotericin B; Invitrogen, Paisley, U.K.). An additional androgen-resistant PC cell line, LN-Flu, was generated in-house by culturing LNCaP cells in RPMI-1640 medium supplemented with 10% FBS and gradually increasing concentrations of 2-hydroxyflutamide up to 2 μM over more than nine months.

Cells were cultured at 37°C in a humidified atmosphere containing 5% CO_2_. *Mycoplasma* contamination was routinely tested using PCR-based methods. For treatment experiments, cells were seeded onto the appropriate culture surface and allowed to adhere for 24 h. Afterwards, the medium was replaced with serum-free RPMI-1640, and treatments were applied as indicated.

### siRNA transfections

siRNA transfection was performed in 1 ml Opti-MEM using Lipofectamine iMAX (4 μl; Invitrogen, Carlsbad, CA, U.S.A.) and 25 nM DPP4-specific siRNA duplexes (5′-GCGUGAAUGAUAAAGGGUtt-3′ and 5′-AGCCCUUUAUCAUUCACGtg-3′) (Ambion, Life Technologies, Carlsbad, CA, U.S.A.), or scrambled control siRNA, according to the manufacturer’s protocol (Invitrogen). Cells were collected at 72 h post-transfection for Western blot analysis.

### Lentiviral transduction for DPP4 and AMPK overexpression

Overexpression of AMPK, DPP4, and mutant DPP4 in PC cell lines was achieved via lentiviral transduction. Lentiviral particles were produced in HEK293T cells by co-transfecting the cells with helper plasmids and the transfer plasmid of interest. The transfection mixture included 5 μg psPAX2, 3 μg pCMV-VSV-G, and 10 μg of the corresponding transfer plasmid (Lenti-TetON-AMPK, pLEX307-DPP4-puro, or pLEX307-DPP4-S630A-puro, as appropriate), and polyethyleneimine (PEI, 1 mg/ml), using a 3:1 PEI:DNA ratio based on the total DNA concentration. Six hours after transfection, the medium was replaced with fresh complete medium. Viral supernatants were collected at 48 and 72 h post-transfection, filtered through a 0.45 μm pore-size filter to remove cell debris, and concentrated by ultracentrifugation at 72,000×***g*** for 90 min at 4°C. After centrifugation, the supernatant was completely removed, and viral particles were allowed to resuspend in the residual volume for 3 h at 4°C.

For transduction, PC cells were seeded in six-well plates and cultured until reaching 100% confluence. Cells were then incubated with concentrated lentiviral particles supplemented with 1 μg/ml polybrene (Sigma–Aldrich, St. Louis, MO, U.S.A.) to enhance transduction efficiency. After a 2-h incubation period, the viral supernatant was diluted by adding fresh culture medium to promote cell recovery. Twenty-four hours post-infection, cells were trypsinized and reseeded in larger culture vessels. One day post-transduction, cells infected with Lenti-TetON-AMPK were selected with 10 mg/ml blasticidin (Fisher Bioreagents, Thermo Fisher Scientific, Pittsburgh, PA, U.S.A.), while those transduced with pLEX307-DPP4-puro or pLEX307-DPP4-S630A-puro were selected using 2 μg/ml puromycin (STEMCELL Technologies, Vancouver, BC, Canada). Selection proceeded for a sufficient duration to enrich for stably infected populations. Finally, Dox was added and maintained for 48 h to induce AMPK overexpression.

### Proteomic analysis

To investigate the impact of DPP4 overexpression on the proteome of PC cells, we conducted a comparative proteomic study using wild-type PC3 cells and PC3 cells stably transduced with the pLEX307-DPP4-puro vector. The experiment was performed in two independent runs, each including six biological replicates per condition.

Cells were seeded in P100 dishes at a density of 1 × 10^6^ cells per plate. After 24 h, cells were washed with PBS, detached using trypsin, and pelleted by centrifugation (300×***g***, 5 min). Supernatants were discarded, and cell pellets were snap-frozen and stored at –80°C until protein extraction. Cell lysis was carried out using trifluoroacetic acid (TFA, ≥99%; Thermo Fisher Scientific, Rockford, IL, U.S.A.), followed by vigorous vortexing to ensure complete disruption. Lysates were neutralized by the addition of eight volumes of 2 M Tris base, and total protein content was determined using the Lowry assay [21].

For mass spectrometry analysis, 4 μg of protein per sample was transferred to a 96-well plate and adjusted to 50 μl with 0.1 M Tris base. Proteins were reduced with 10 mM tris(2-carboxyethyl)phosphine and alkylated with 40 mM chloroacetamide, followed by overnight digestion with trypsin at 37°C (10 ng/μg protein). Resulting peptides were purified using C18 stage tips, prepared by stacking five C18 discs into 200-μl pipette tips. Tips were conditioned with 250 μl acetonitrile, washed with solvent B (40% acetonitrile, 0.5% acetic acid, 60% water), and equilibrated with solvent A (0.5% acetic acid in Milli-Q water), each step followed by centrifugation at 2700×***g*** for 5 min. Peptides were acidified to pH 2–3 with TFA, loaded onto the tips, washed, and eluted with 40 μl of solvent B. Eluates were dried under vacuum and stored at –20°C. Before LC-MS/MS, samples were reconstituted in 40 μl of solvent A. Peptide separation was performed on a 25-cm C18 reverse-phase column (nanoElute 2, Bruker Daltonics) using a 30-min linear gradient, as previously described [22].

Mass spectrometry analysis was carried out on a timsTOF fleX instrument (Bruker Daltonics) operating in library-free data-independent acquisition (DIA) mode with parallel accumulation–serial fragmentation. Protein identification was performed using FragPipe v22.0 [23,24], searching against the UniProt-reviewed human proteome (UP000005640; downloaded December 2, 2024). Quantification was conducted with DIA-NN v1.9 [25], with identifications filtered at a stringent false discovery rate (FDR) of <1%. Label-free quantification was normalized using the MaxLFQ algorithm [26], as implemented in FragPipe.

Bioinformatic and statistical analyses were performed using FragPipe-Analyst (https://fragpipe-analyst.org/) and R (v4.4.2). Proteins with ≥50% non-missing values in at least one condition (*n* = 6) were retained. Abundance values were normalized using variance stabilizing normalization, and missing values were imputed using a Perseus-like strategy.

Subsequent analyses were based on log_2_-transformed LFQ intensities. Differential expression was assessed using ANOVA and pairwise moderated *t*-tests with FDR correction. Downstream analyses were conducted in R using custom scripts adapted from publicly available resources. The tidyverse, pheatmap, clusterProfiler [27,28], BiocManager, and enrichplot [29] packages were employed for differential expression analysis, visualization (Volcano plots and heatmaps), and functional enrichment (Gene Ontology (GO) and Kyoto Encyclopedia of Genes and Genomes (KEGG)).

### Wound-healing assay

Cells were seeded in six-well plates at a density of 6 × 10^5^ cells per well. After three days, upon reaching full confluence, fresh medium was added, and a linear scratch (‘wound’) was created using a cell scraper. An initial reference image was captured to document the 0 h time point. To ensure consistent imaging throughout the assay, two alignment marks were drawn on the underside of each well flanking the scratch area. Images of the wound were taken at the indicated time points using the same reference coordinates. When required, the culture medium was replenished with fresh medium to maintain optimal cell viability. Wound closure was quantified using ImageJ software by measuring the wound area at each time point under the different experimental conditions.

### Cell proliferation assay using CFSE staining

Cell proliferation was evaluated using the BioTracker™ 488 Green CFSE Cell Proliferation Kit (Millipore, Merck, Darmstadt, Germany). Briefly, 2 × 10^6^ cells were harvested by centrifugation at 300×***g*** for 5 min and resuspended in 2 ml of pre-warmed PBS (37°C). CFSE (carboxyfluorescein succinimidyl ester) was added at a final concentration of 1 μM (2 μl of a 2.5 μM stock per 2 ml), and cells were incubated for 15 min at 37°C in the dark to facilitate intracellular dye incorporation. To quench unbound dye, 2 ml of complete culture medium was added, followed by a 5-min incubation at 37°C. After centrifugation, cells were resuspended in 2 ml of fresh complete medium and incubated for an additional 30 min at 37°C to ensure covalent binding of the dye to intracellular proteins. Cells were then washed, resuspended in fresh medium, and stained with propidium iodide to exclude dead cells. Baseline fluorescence (time 0 h) was recorded using a MACSQuant® Analyzer flow cytometer (Miltenyi Biotec, Bergisch Gladbach, Germany). Subsequently, 3 × 10^5^ labeled cells were seeded per well in 6-well plates. After three days of culture, the medium was replaced and cells were treated with CAP (80 or 160 μM) for 48 h. Fluorescence intensity was measured by flow cytometry, and data were analyzed using MACSQuantify software (Miltenyi Biotec).

### Animal studies

Twelve male TRAMP (Transgenic Adenocarcinoma of the Mouse Prostate) mice (C57BL/6-Tg(TRAMP)8247Ng/J × FVB/NJ)F1/J) were obtained from The Jackson Laboratory (Bar Harbor, ME, U.S.A.). Animals were housed and cared for at the Animal Experimentation Center (CEA) of the University of Alcalá (registration number ES280050001165), which guarantees compliance with established standards for animal welfare, care, and maintenance throughout the duration of the study). Upon arrival at 4–6 weeks of age, mice were randomly assigned to one of two dietary groups (*n* = 6 per group): a standard diet (STD) or a standard diet supplemented with capsaicin (STD + CAP). Both diets were supplied by the Central Research Services of the University of Almería (Spain) and were based on the AIN-93M maintenance formulation. For the CAP-enriched diet, 0.01% CAP was added to the standard formulation, a dose equivalent to the average daily intake of CAP in mild U.S.A. and European diets (approximately 1.5 mg/person/day). The complete dietary composition is detailed in Table 1.

**Table 1:** Composition of the experimental diets used in the present study

	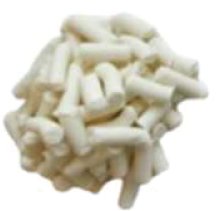	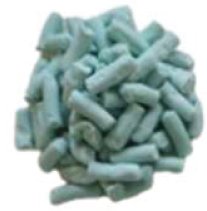
Ingredients (g)	STD	STD + CAP
Casein	140.0	140
L-cysteine	1.8	1.8
Maize starch	465.7	465.7
Maltodextrin	155	155
Sucrose	100	100
Cellulose	50	50
Soya bean oil	20	20
Pork fat	20	20
BHQ	0.008	0.008
Minerals	35	35
Vitamins	10	10
Choline bitartrate	2.5	2.5
Capsaicin		0.1
For 1 kg of mixture	1000.0	1000.1

The table outlines the formulation of the standard AIN-93M control diet and its corresponding variant supplemented with 0.1 g/kg CAP.

Mice were housed in a temperature-controlled facility (24°C) under a 14/10-h light/dark cycle with *ad libitum* access to food and water. To ensure animal welfare, they were kept in groups of three per cage with cork dust bedding and continuous environmental enrichment. Body weights were monitored every three days throughout the 24-week dietary intervention.

At the end of the experimental period, animals were anesthetized in an induction chamber with 4% isoflurane, followed by maintenance anesthesia at 0.5% in oxygen. While under deep anesthesia, mice were exsanguinated by cardiac puncture. After completion of blood collection, animals were killed by carbon dioxide inhalation. Prostate tissues were collected immediately post-mortem for downstream analyses.

### Protein extraction and western blot analysis

Prostate tissues from mice were mechanically dissociated using a sterile scalpel and subsequently homogenized with a Polytron PT 3100 homogenizer (Kinematica, Luzern, Switzerland) in the presence of lysis buffer (50 mM Tris-HCl, pH 7.4, 0.8 M NaCl, 5 mM MgCl_2_, 0.1% Triton X-100) supplemented with protease and phosphatase inhibitor cocktails (Roche Diagnostics, Mannheim, Germany). Samples were sonicated on ice (3 s, 25% amplitude) with a Sonifier Cell Disruptor B15 (Branson, Danbury, CT, U.S.A.). Proteins of cell samples were extracted by resuspending the samples in lysis buffer. Subsequently, all the samples (tissue and cells) were incubated on ice for 15 min, and centrifuged (15,870×***g***, 10 min, 4°C) to remove debris. Protein concentration was determined using the Bradford Protein Assay Kit (Bio-Rad, Hercules, CA, U.S.A.). For Western blotting, 20 μg of total protein was separated by SDS-PAGE and transferred onto PVDF membranes. Membranes were incubated overnight at 4°C with primary antibodies (Table 2), followed by secondary antibodies (1:5000) for 2 h at room temperature. Immunoreactions were visualized using ClarityTM Western ECL Substrate (Bio-Rad, Hercules, CA, U.S.A.) and detected with a ChemiDoc MP Imaging System (Bio-Rad, Hercules, CA, U.S.A.). Protein expression levels were quantified using ImageJ (NIH, Bethesda, MD, U.S.A.) and expressed as fold changes relative to controls. All Western blots were performed in triplicate.

**Table 2:** List of antibodies employed in Western blotting, including corresponding dilutions, catalog numbers, and suppliers

Antibody	Dilution	Reactivity	Reference and source
DPP4	1:1000	Human	67138; Cell Signaling Technology (Danvers, MA, U.S.A.)
DPP4	1:1000	Mouse	61408; Cell Signaling Technology (Danvers, MA, U.S.A.)
AR	1:500	Human	sc-7305; Santa Cruz Biotechnology (TX, U.S.A.)
PSA	1:1000	Human and mouse	PA5-120742, Invitrogen (Carlsbad, CA, U.S.A.)
PSMA	1:1000	Human	12815; Cell Signaling Technology (Danvers, MA, U.S.A.)
pACC	1:1000	Human and mouse	3661; Cell Signaling Technology (Danvers, MA, U.S.A.)
ACC	1:1000	Human and mouse	3662; Cell Signaling Technology (Danvers, MA, U.S.A.)
E-cadherin	1:1000	Human	3195; Cell Signaling Technology (Danvers, MA, U.S.A.)
N-cadherin	1:500	Human	14215; Cell Signaling Technology (Danvers, MA, U.S.A.)
EpCAM	1:1000	Human	42515, Cell Signaling Technology (Danvers, MA, U.S.A.)
β-Actin	1:5000	Human and mouse	A5441; Sigma–Aldrich (St. Louis, MO, U.S.A.)
HRP anti-mouse IgG	1:5000	Mouse	A-9044; Sigma–Aldrich (St. Louis, MO, U.S.A.)
HRP anti-rabbit IgG	1:5000	Rabbit	#7074S; Cell Signaling Technology (Danvers, MA, U.S.A.)

### RNA extraction and quantitative PCR

Total RNA was extracted from cultured cells using the NZY Total RNA Isolation Kit (NZYTech, Lisbon, Portugal) following the manufacturer’s instructions. Subsequently, 2 μg of RNA was reverse-transcribed into cDNA using the NZY First-Strand cDNA Synthesis Kit (NZYTech, Lisbon, Portugal). Quantitative real-time PCR (qPCR) was performed in 10 μl reaction volumes using NZYSpeedy qPCR Green Master Mix (2×) ROX, on a QuantStudio™ 3 Real-Time PCR System (Thermo Fisher Scientific, Waltham, MA, U.S.A.). Gene-specific primers used for amplification are listed in Table 3.

**Table 3:** Oligonucleotide sequences employed for qPCR amplification in human and murine specimens

Gene (mouse)	Forward (5′–3′)	Reverse (5′–3′)
*DPP4*	5′-ACCGTGGAAGGTTCTTCTGG-3′	5′-CACAAAGAGTAGGACTTGACCC-3′
*Oct4*	5′-CGGAAGAGAAAGCGAACTAGC-3′	5′-ATTGGCGATGTGAGTGATCTG-3′
*Nanog*	5′-CACAGTTTGCCTAGTTCTGAGG-3′	5′-GCAAGAATAGTTCTCGGGATGAA-3′
*AR*	5′-TGTGGAGATGAAGCTTCTGGCTGT-3′	5′-TGGTACAATCGTTTCTGCTGGCAC-3′
*PSMA*	5′-GCCTTACAACGTGGGACCTG-3′	5′-CATCTCGGTGACCTCCAAGAA-3′
*GAPDH*	5′-TGAAGCAGGCATCTGAGGG-3′	5′-CGAAGGTGGAAGAGTGGGAG-3′
*AMPKα1*	5′-TGCTACTCCACAGAGATCGG-3′	5′-GTCTGAGGGCTTTCCTTGAG-3′
*AMPKα2*	5′-CGACTACATCTGCAAACATGG-3′	5′-CAGTAATCCACGGCAGACAG-3′

### Statistical analysis

Statistical analyses, unless otherwise specified in the proteomics section, were performed using GraphPad Prism version 9.0 (GraphPad Software, San Diego, CA, U.S.A.). Two-way ANOVA followed by Tukey’s or Sidak’s multiple comparisons test was used, as appropriate. Data are presented as mean ± standard deviation (SD) or standard error when indicated, from at least three independent experiments, as indicated in the corresponding figure legends. Differences were considered statistically significant at *P*≤0.05.

## Results

### Proteomic analysis reveals a role for DPP4 in modulating cell adhesion pathways in prostate cancer cells

To explore the functional role of DPP4 in PC, we conducted a quantitative proteomic analysis using androgen-resistant PC3 cells given that they are a well-established, canonical model of androgen-independent and highly aggressive PC. DPP4 overexpression was induced via lentiviral transduction with a pLEX307-DPP4-puro construct as detailed in the Methods section. Overexpression of DPP4 was confirmed by Western blot (Supplementary Figure S2).

**Figure 1:**
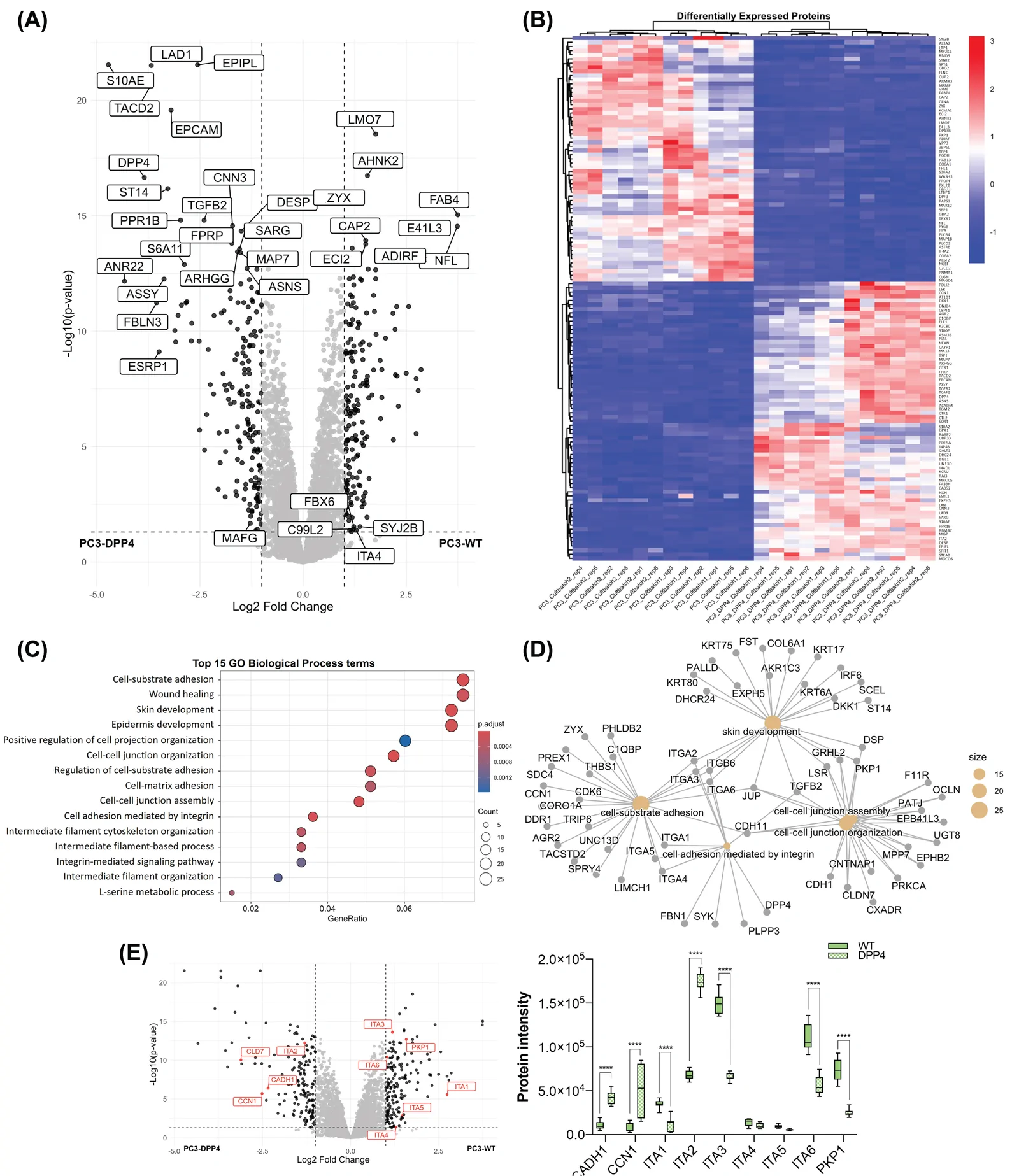
Proteomic profiling of wild-type and DPP4-overexpressing PC3 cells (**A**) Volcano plot depicting DEPs between unmodified PC3 cells (PC3-WT) and PC3 cells overexpressing DPP4 via pLEX307-DPP4-puro transduction (PC3-DPP4). Proteins exhibiting a log_2_ fold change ≥ |1| with *P<*0.05 are highlighted in black. (**B**) Heatmap illustrating DEPs with a log_2_ fold change of ≥|1.2|, with hierarchical clustering applied to both proteins and sample groups. (**C**) Enrichment analysis of GO biological processes among the DEPs. The top 15 significantly enriched terms are displayed. Dot size corresponds to the number of proteins involved, and color gradient reflects statistical significance (adjusted *P*-value). (**D**) Network representation of enriched biological processes linked to the DEPs. GO terms are represented as yellow nodes, and associated DEPs are shown as gray nodes. (**E**) Left: Volcano plot identifying adhesion-associated proteins in red. Statistically significant DEPs (log_2_ fold change ≥ |1|, *P<*0.05) are marked in black. Right: Boxplots showing expression levels of the highlighted adhesion proteins. WT samples are depicted as solid green bars, while DPP4-overexpressing cells are shown with striped green bars. Significance was assessed using two-way ANOVA followed by Sidak’s post hoc test: *P<0.05; **P<0.01; ***P<0.001; ***P<0.0001.

Proteomic profiling was performed to identify differentially expressed proteins (DEPs) between DPP4-overexpressing cells (PC3-DPP4) and wild-type PC3 cells (PC3-WT). Applying a log_2_ fold change ≥ |1| and a significance level of *P<*0.05, we identified a total of 350 DEPs, with 175 up-regulated in the PC3-WT group and 175 enriched in PC3-DPP4 cells. The distribution of DEPs is illustrated in the volcano plot (Figure 1A), while hierarchical clustering of the samples based on these DEPs is shown in the heatmap (Figure 1B; enlarged in Supplementary Figure S3), highlighting clear expression differences between the experimental groups.

To gain insight into the biological relevance of these DEPs, we performed GO enrichment analysis. The most significantly enriched biological processes were associated with cell adhesion, including cell–substrate adhesion, wound healing, skin development, and epidermis development (Figure 1C). Network-based analysis (Figure 1D) highlighted the central role of adhesion-related processes, specifically cell–substrate adhesion, cell–adhesion mediated by integrins, cell–cell junction organization and assembly, and skin development. Complementary KEGG pathway analysis (Supplementary Figure S4) also revealed significant alterations in amino acid biosynthesis and metabolism, particularly involving glycine, serine, and threonine, arginine and proline, and, to a lesser extent, alanine, aspartate, and glutamate.

To specifically assess the impact of DPP4 overexpression on cell adhesion mechanisms, we examined the expression levels of selected proteins involved in this process. Quantitative analysis of protein intensities (Figure 1E, left panel) revealed increased expression of E-cadherin, CCN1, Claudin-7, and integrin alpha-2 in PC3-DPP4 cells. In contrast, PC3-WT cells exhibited higher levels of integrin alpha-1, -3, -4, -5, and -6, as well as plakophilin-1. These findings suggest that DPP4 overexpression promotes a phenotypic shift toward a more epithelial and less mesenchymal state, supporting a potential role for DPP4 in modulating cell adhesion processes in PC.

### DPP4 Overexpression in prostate cells restores AR signaling and suppresses prostate cancer cell migration and proliferation

**Figure 2:**
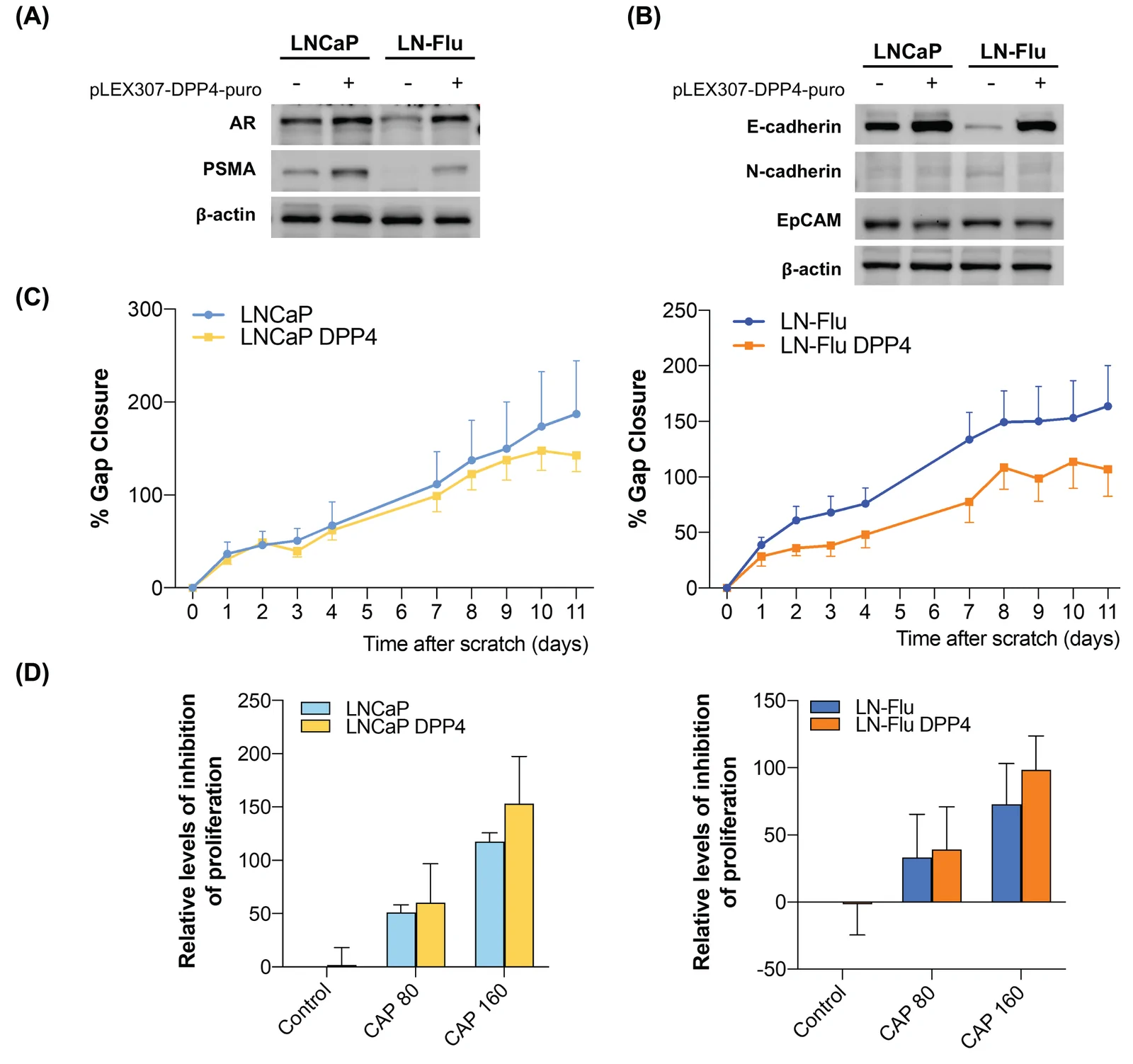
Effects of DPP4 overexpression in androgen-sensitive and -insensitive PC cells LNCaP and LN-Flu cells were transduced with the pLEX307-DPP4-puro vector to induce DPP4 overexpression. (**A**) Western blot analysis of AR and PSMA expression following DPP4 overexpression in LNCaP and LN-Flu cells. β-Actin was used as a loading control. (**B**) Western blot analysis of E-cadherin, N-cadherin, and epithelial cell adhesion molecule (EpCAM) expression, markers involved in cell adhesion. (**C**) Quantification of wound closure in LNCaP and LN-Flu cells overexpressing DPP4, represented as percentage closure. Data are shown as mean ± SE (*n* = 3). (**D**) Relative inhibition of cell proliferation in LNCaP and LN-Flu cells overexpressing DPP4 after 24-h treatment with 80 or 160 μM CAP. Cell proliferation was assessed using the CFSE assay, and fluorescence intensity was measured by flow cytometry. Relative inhibition was calculated by comparing fluorescence levels between treated and untreated cells. Data are presented as mean ± SD (*n* = 3).

To further investigate the function of DPP4 and its involvement in prostate cell adhesion and migration, we compared the androgen-sensitive LNCaP cells overexpressing DPP4 with the androgen-resistant LN-Flu cells, which are adapted to growth in the presence of a high concentration of the antiandrogen 2-hydroxy-flutamide, overexpressing DPP4. As shown in Figure 2A, the overexpression of DPP4 in LNCaP and LN-Flu cells resulted in an increase in AR levels and its target gene, PSMA, reinforcing the notion that DPP4 overcomes androgen resistance, as previously suggested [11,14].

The loss of cell adhesion is a critical step in the metastatic cascade, enabling tumor cells to detach from the primary tumor and invade distant tissues. A hallmark of this process is the down-regulation of E-cadherin, a key epithelial cell–cell adhesion molecule, which disrupts adherent junctions and promotes epithelial-to-mesenchymal transition (EMT). Concurrently, the up-regulation of N-cadherin, typically expressed in mesenchymal cells, facilitates enhanced motility and invasive capabilities. To further investigate the role of DPP4 in cell adhesion and motility, we analyzed the expression of key cell adhesion markers. As shown in Figure 2B, DPP4 overexpression led to an up-regulation of E-cadherin and a down-regulation of N-cadherin, indicative of enhanced cell–cell adhesion and suppression of epithelial-to-mesenchymal transition, which correlates with the proteomic results.

We additionally determined the expression of the EpCAM, a transmembrane glycoprotein that facilitates homotypic cell–cell adhesion in epithelial tissues. Beyond its role in adhesion, EpCAM also contributes to cell signaling, migration, proliferation, and differentiation. EpCAM is overexpressed in 40%–60% of PCs and is associated with metastasis, increased risk of PC recurrence, and resistance to treatment [30]. Our results show that DPP4 overexpression decreases the levels of EpCAM, suggesting an inhibition of migration and proliferation.

To corroborate the impact of DPP4 on cell motility and adhesion, we performed a wound healing assay. Our results showed that DPP4 overexpression significantly reduced both the migratory and invasive capabilities of LNCaP and LN-Flu cells compared with control cells (Figure 2C), reinforcing the regulatory role of DPP4 in tumor progression. Then, we determined cell proliferation by the CFSE proliferation assay, which monitors cell division and proliferation by labeling cells with a fluorescent dye, which becomes diluted with each cell division. Results in Figure 2D show that DPP4 overexpression not only inhibited the proliferation of LNCaP and LN-Flu cells but also potentiated the inhibitory effect of the antitumoral natural agent CAP.

### Effect of catalytically inactive DPP4 on AR and adhesion markers expression

**Figure 3:**
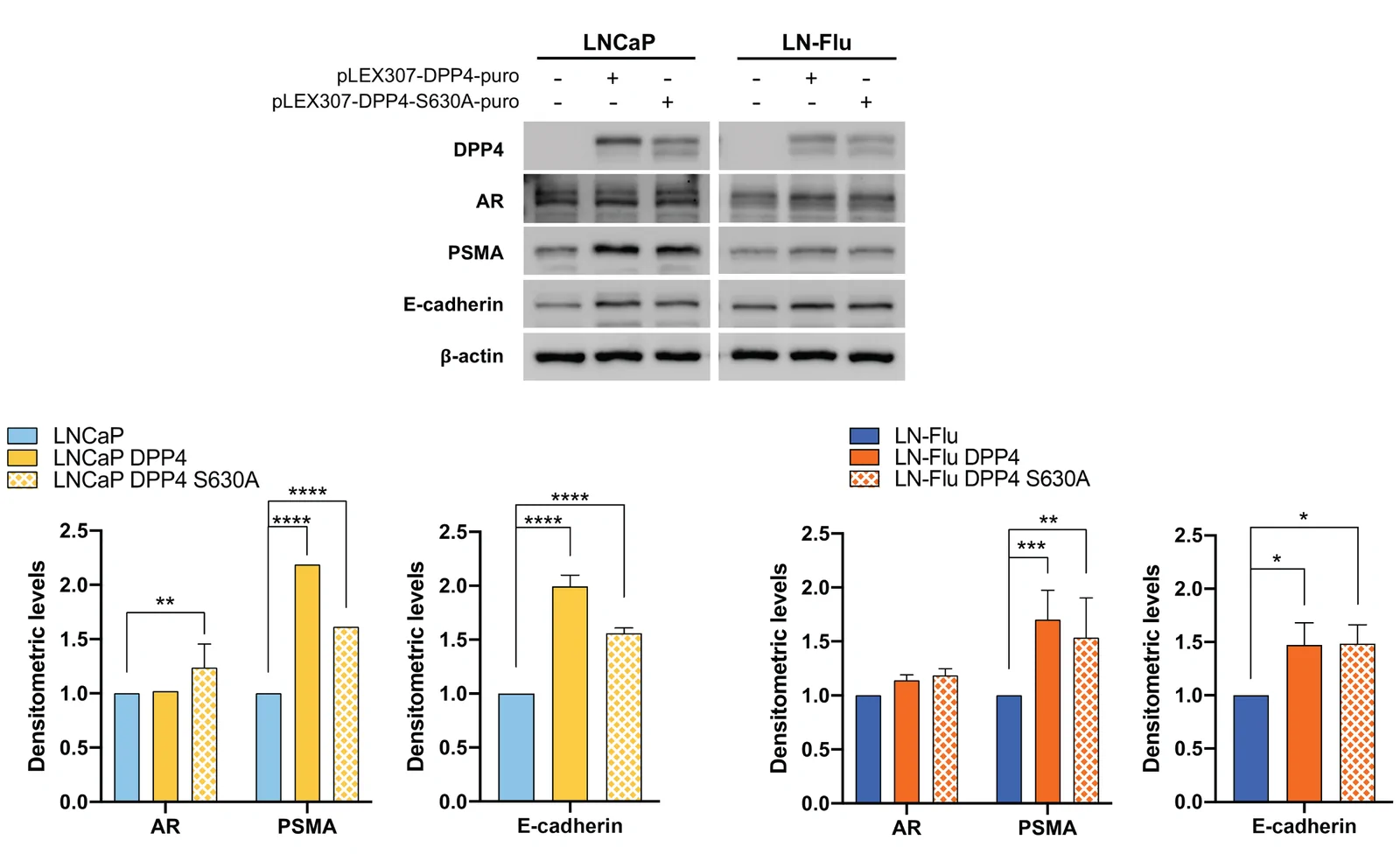
Effects of mutant (S630A) DPP4 overexpression in androgen-sensitive and androgen-insensitive PC cells LNCaP and LN-Flu cells were transduced with the pLEX307-DPP4-S630A-puro vector to induce mutant DPP4 overexpression. Western blot analysis of DPP4, AR, PSMA, and E-cadherin expression was performed in LNCaP and LN-Flu cells following mutant DPP4 overexpression. Representative immunoblots and the corresponding densitometric analyses are shown for each protein. β-Actin was used as the loading control. Data are presented as the mean ± SD (*n* = 3). Statistical significance was determined by two-way ANOVA followed by Tukey’s multiple comparisons test. *P<*0.05 (*), *P<*0.01 (**), *P<*0.001 (***), and *P<*0.0001 (****).

To further investigate the involvement of DPP4 in the restoration of AR signaling, we generated a catalytically inactive DPP4 mutant containing a substitution of the active-site residue Ser630 by alanine (S630A). LNCaP and LN-Flu cells were transduced with a lentiviral vector encoding the mutant DPP4 construct to achieve stable overexpression (Figure 3). We then evaluated the expression of AR, its target gene PSMA, and the epithelial adhesion marker E-cadherin. As shown in Figure 3, overexpression of the S630A DPP4 mutant, despite lacking catalytic activity, still increased the expression of AR, PSMA, and E-cadherin. These findings indicate that the effects of DPP4 on AR restoration and epithelial marker expression are independent of its enzymatic activity and are more likely mediated by a non-catalytic scaffolding or chaperone-like function.

### DPP4 silencing reverses the effects of DPP4 overexpression on AR expression and cell adhesion markers

**Figure 4:**
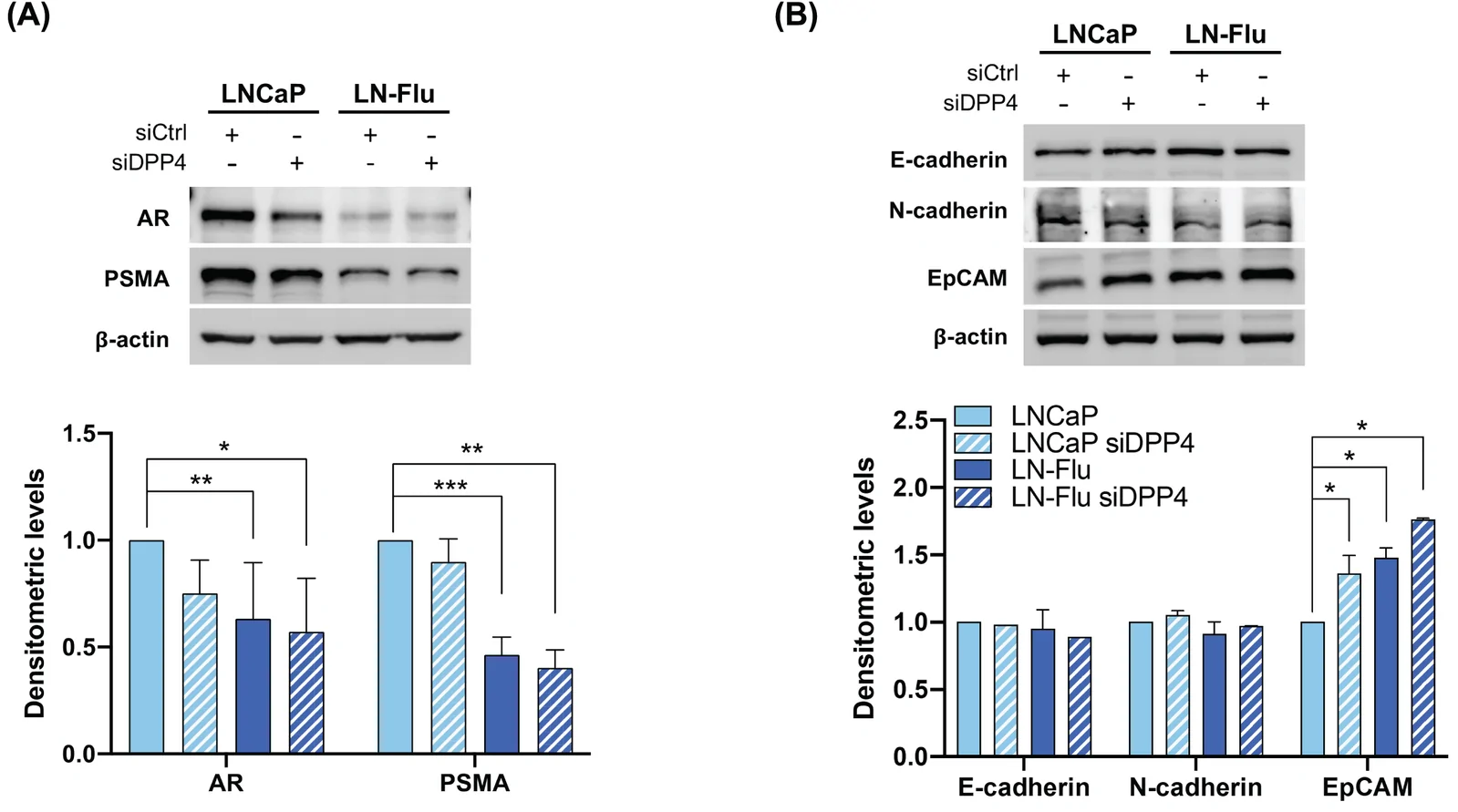
Effects of DPP4 silencing in androgen-sensitive and androgen-insensitive PC cells (**A**) Western blot analysis of AR and PSMA expression and (**B**) E-cadherin, N-cadherin, and EpCAM expression following DPP4 silencing in LNCaP and LN-Flu cells. Representative immunoblots and the corresponding densitometric analyses are shown for each protein. β-Actin was used as the loading control. Data are presented as the mean ± SD (*n* = 3). Statistical significance was determined by two-way ANOVA followed by Tukey’s multiple comparisons test. *P<*0.05 (*), *P<*0.01 (**), and *P<*0.001 (***).

To further investigate the role of DPP4 in androgen receptor (AR) restoration, DPP4 expression was silenced in LNCaP and LN-Flu cells using siRNA. Although both cell lines exhibit low basal DPP4 expression, siRNA-mediated knockdown further reduced DPP4 levels (Supplementary Figure S5). Interestingly, DPP4 knockdown not only prevented the DPP4-mediated restoration of AR expression but also further decreased AR protein levels in both cell lines (Figure 4 A). Likewise, the AR target gene PSMA decreased when DPP4 was silenced (Figure 4A), reinforcing the notion that DPP4 collaborates in AR expression and function restoration in PC cells.

We next examined the expression of cell adhesion markers following DPP4 silencing. As shown in Figure 4B, in contrast with DPP4 overexpression, DPP4 knockdown failed to increase E-cadherin expression in LNCaP cells and slightly reduced its expression in LN-Flu cells, thereby abolishing the EMT-suppressive effect observed upon DPP4 overexpression. Likewise, DPP4 knockdown did not reduce N-cadherin expression in either LNCaP or LN-Flu cells (Figure 4B). These findings indicate that DPP4 contributes to the inhibition of EMT observed following DPP4 overexpression. In contrast, DPP4 knockdown induced an increase in the metastasis-associated glycoprotein EpCAM both in LNCaP and LN-Flu cells (Figure 4B), further supporting the role of DPP4 in suppressing invasiveness.

To further validate these findings, DPP4 was silenced in DPP4-overexpressing cells (Supplementary Figure S6), as endogenous DPP4 expression is too low in the parental cell line to permit efficient knockdown. As shown in Supplementary Figure S6, DPP4 silencing confirmed its role in maintaining AR signaling and suppressing EMT. Specifically, knockdown of DPP4 in DPP4-overexpressing cells resulted in a slightly reduced expression of AR and PSMA (Supplementary Figure S6A). Moreover, DPP4 silencing, although not significantly, decreased E-cadherin expression while increasing the levels of N-cadherin and EpCAM (Supplementary Figure S6B), indicating a reversal of the epithelial phenotype. Collectively, these results demonstrate that DPP4 down-regulation abrogates the DPP4-mediated restoration of AR signaling and its inhibitory effect on EMT, even in cells ectopically expressing DPP4.

### DPP4 Down-regulation in castration-resistant cells is reversed by capsaicin

**Figure 5:**
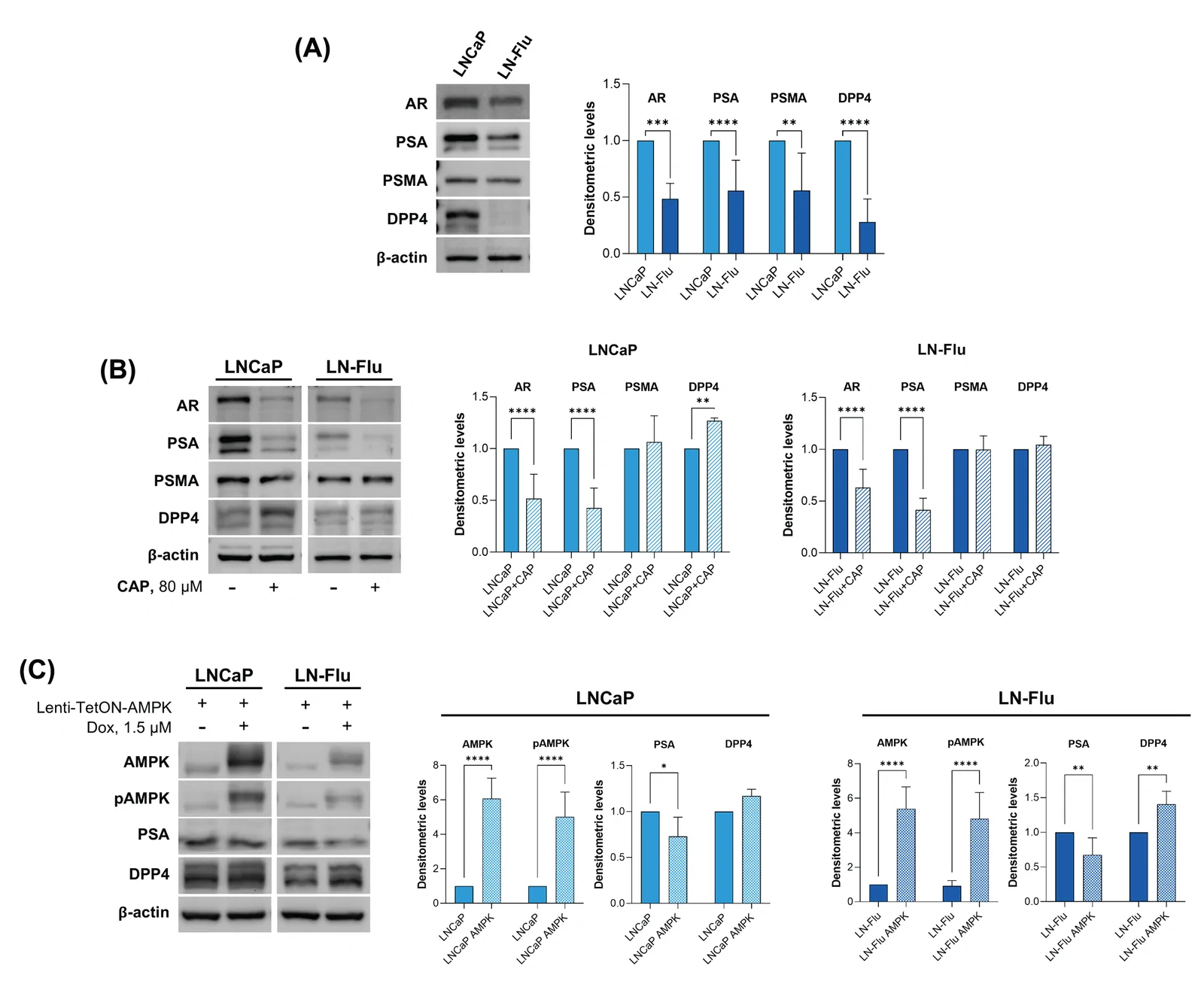
Expression of AR, PSA, PSMA, and DPP4 in hormone-sensitive and -resistant PC cells, and modulation by CAP treatment (**A**) Basal protein levels of AR, PSA, PSMA, and DPP4 in hormone-sensitive LNCaP and hormone-resistant LN-Flu PC cells, as determined by Western blot analysis. Densitometric quantification (mean ± SD), normalized to LNCaP, is shown on the right. (**B**) Effect of CAP treatment (80 μM, 24 h) on AR, PSA, PSMA, and DPP4 expression in LNCaP and LN-Flu cells. Protein levels were assessed by Western blotting. β-Actin was used as a loading control (*n* = 3). Densitometric quantification (mean ± SD), normalized to untreated LNCaP or LN-Flu cells, is shown on the right. (**C**) Effect of AMPK overexpression on PSA and DPP4 protein levels. LNCaP and LN-Flu cells were transduced with a Dox-inducible Lenti-TetON-AMPKα1β1γ1 construct. Overexpression was induced with 1.5 μM Dox for 48 h. Protein levels were analyzed by Western blotting; GAPDH served as a loading control (*n* = 3). Densitometric quantification (mean ± SD), normalized to LNCaP or LN-Flu cells not treated with Dox, is shown on the right. Statistical significance: *P<*0.05 (*), *P<*0.01 (**), *P<*0.001 (***), and *P<*0.0001 (****); two-way ANOVA and Sidak’s multiple comparisons test.

To investigate the role of DPP4 in androgen resistance of prostate cells, we compared the androgen-sensitive prostate LNCaP cell line with the androgen-resistant LN-Flu cell line. As seen in Figure 5A, levels of the AR were lower in the androgen-resistant cell line (LN-Flu) than in the androgen-sensitive LNCaP cell line. Similarly, levels of the PSA and the prostate-specific membrane antigen (PSMA), two well-known AR-regulated genes, were lower in LN-Flu cells. Interestingly, the levels of DPP4 were also lower in LN-Flu cells compared with LNCaP cells, reinforcing the notion that DPP4 is an androgen-regulated gene that decreases in the transition to castration resistance.

CAP, a homovanillic acid derivative (8-methyl-*N*-vanillyl-6-nonenamide) and the active component of red peppers (genus *Capsicum*), has demonstrated antiproliferative properties against PC cells by inducing apoptosis, inhibiting cell cycle progression, and modulating key signaling pathways [31–34]. We have previously shown that, depending on the dose, CAP modulates the expression of the AR in LNCaP cells [35]. Then, we hypothesized that CAP may play a role in modulating DPP4 activity or expression. To evaluate this, LNCaP and LN-Flu cells were treated with 80 μM CAP for 24 h. It is worth noting that although CAP treatment reduced the levels of the AR as well as those of PSA, it increased the expression of DPP4 (Figure 5B), suggesting that CAP overcomes the transition to castration resistance.

We previously demonstrated that CAP’s effect on PC is mediated by AMPK [32,36]. To evaluate the role of AMPK in DPP4 expression, we infected LNCaP and LN-Flu cells with a Dox-inducible lenti-TetON plasmid encoding the α1, β1, and γ1 subunits of AMPK (Lenti-TetON-AMPK) (Supplementary Figure S1). As shown in Figure 5C, Dox induction of AMPK led to increased DPP4 expression, particularly in the resistant LN-Flu cells. This effect was accompanied by a slight decrease in PSA expression in LN-Flu cells (Figure 5C).

### Capsaicin restores DPP4 expression in the TRAMP mouse model of prostate cancer

**Figure 6:**
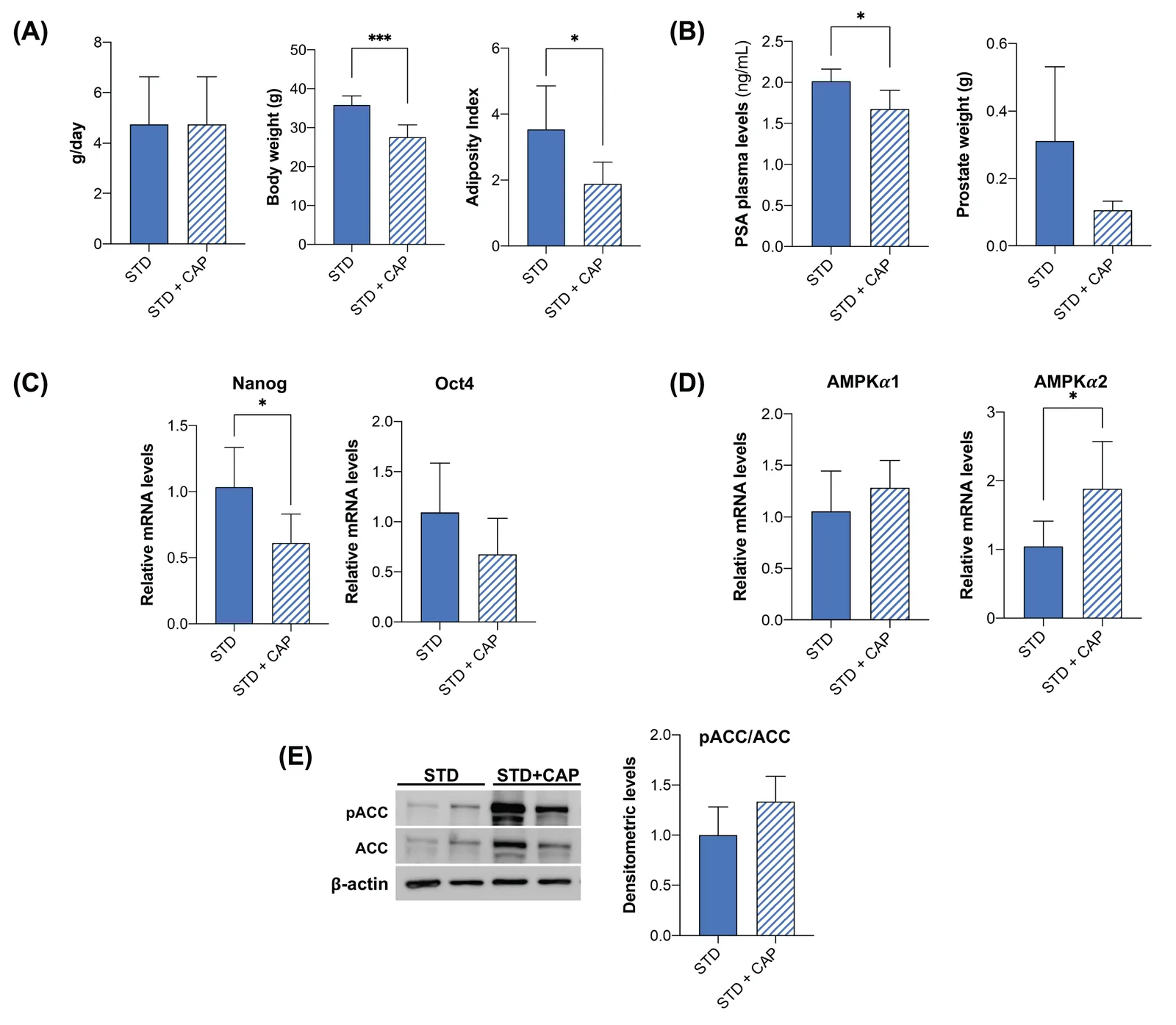
CAP supplementation alters fat accumulation, PSA levels, stemness markers, and AMPK signaling in TRAMP mice (**A**) Total food intake (g/mouse/day, left), final body weight (middle), and adiposity index (right) calculated as: 100 × (mesenteric fat weight + epididymal fat weight + perirenal fat weight)/total body weight. Mice were assigned to an STD group (*n* = 6) or an STD + CAP group (*n* = 6). Data are expressed as the relative change in mean values of the STD + CAP group compared with the STD group. (**B**) Plasma PSA levels (left) and prostate weight (right) at the end of the treatment. PSA levels were quantified by ELISA. (**C**) mRNA expression levels of the cancer stem cell markers Nanog and Oct4 in TRAMP mouse prostates, determined by qPCR. (**D**) Relative expression of AMPKα1 and AMPKα2 in prostate tissue, assessed by qPCR and normalized to GAPDH. Data are shown as mean ± SD. (**E**) Western blot analysis of phosphorylated and total acetyl-CoA carboxylase (ACC) protein levels in prostate tissue. β-Actin was used as a loading control. Representative blots from two animals per group are shown. Densitometric quantification (mean ± SD), normalized to the STD group, is displayed on the right. Data are shown as the mean ± SD; *P<*0.05 (*), *P<*0.01 (**), *P<*0.001 (***) and *P<*0.0001 (****) indicate significant differences by unpaired *t*-test.

To investigate the role of DPP4 in castration-resistant PC and its modulation by CAP, we used the TRAMP model, a widely used genetically engineered mouse used in PC research, which spontaneously develops human-like PC, including metastases [37]. Mice were fed *ad libitum* with either an STD or an STD + CAP for 24 weeks. Daily food intake was comparable between both groups (Figure 6A, left panel), indicating that CAP did not cause diet rejection or induce addiction. However, the final body weight, as well as the adiposity index determined as 100 × (mesenteric fat weight + epididymal fat weight + perirenal fat weight)/body weight, was significantly lower in the CAP-fed mice compared with standard diet-fed animals (Figure 6A, middle and right panels) which point to the effect of CAP on energy expenditure and is in agreement with previous data [38,39].

Interestingly, the PSA plasma levels of the CAP-fed group were lower than those in the STD group (Figure 6B, left panel), which indicates the effect of CAP on prostate proliferation. CAP treatment led to a reduction in prostate weight in TRAMP mice, which are genetically predisposed to develop prostate tumors, providing additional evidence for CAP’s antiproliferative role in prostate tumorigenesis (Figure 6B, right panel). Moreover, we found that CAP down-regulated the expression of the cancer stem cell markers Nanog and Oct4 in the prostate, further reinforcing the antitumor effect of CAP on the prostate (Figure 6C).

As mentioned earlier, we previously reported that CAP treatment activates the AMPK in prostate cells [32,36]. To investigate whether CAP enhanced AMPK activity in the TRAMP mice, we examined the expression of AMPK and the phosphorylation of its well-known substrate, ACC, a key marker of AMPK activation. Interestingly, qPCR analysis revealed a significantly increased expression of the alpha-2 isoform subunit and a slight increase in the alpha-1 subunit of AMPK in CAP-fed animals (Figure 6D). Accordingly, Figure 6E shows an increased expression of both total and phosphorylated forms of ACC in prostate tissue from animals fed a CAP-enriched diet. Consistently, the pACC/ACC ratio was also elevated in the CAP-treated group (Figure 6E, right panel). These results strengthen the notion that supplementation of the diet with CAP activates AMPK in prostate tumors.

**Figure 7:**
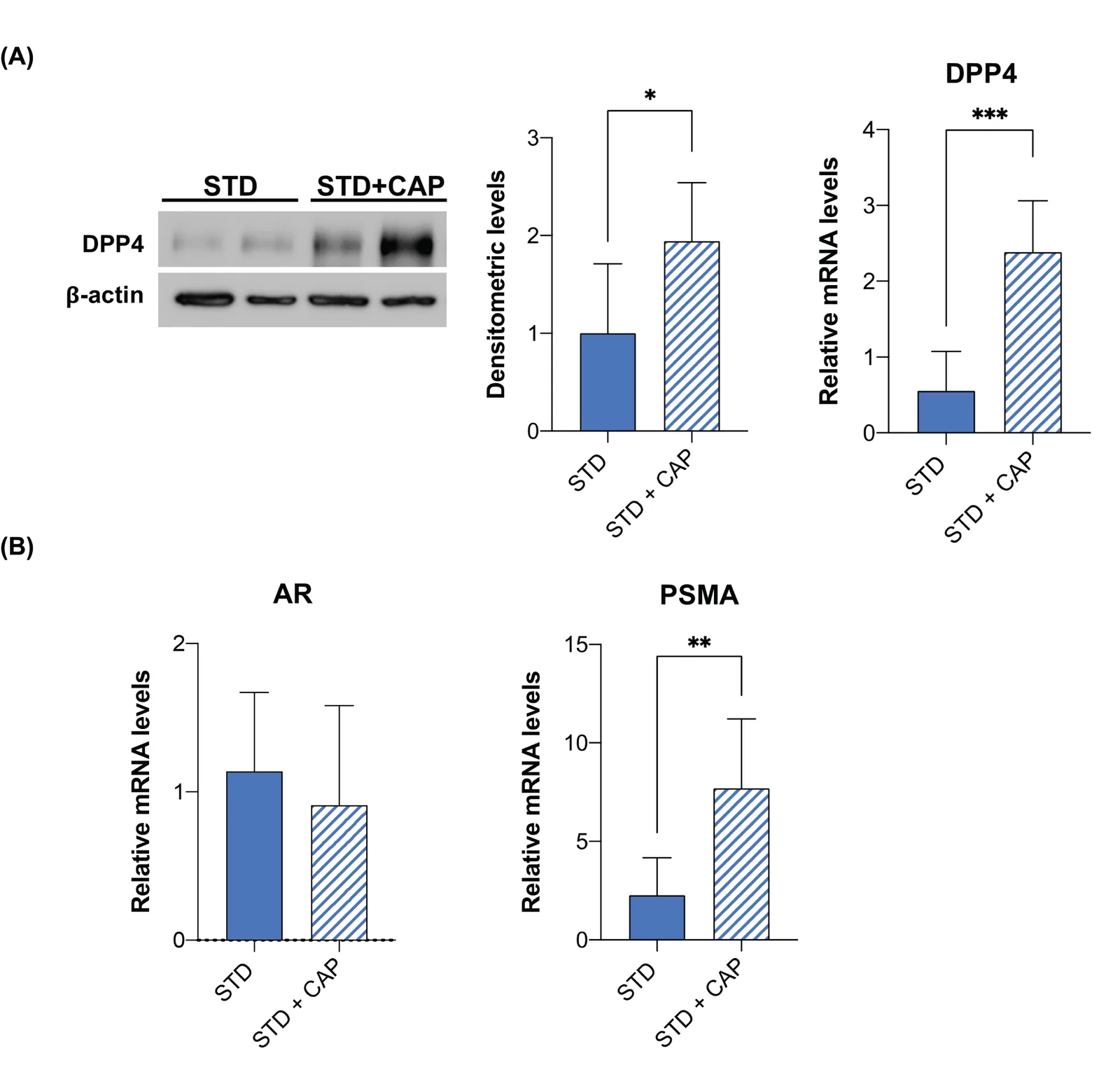
CAP supplementation modulates DPP4, AR and PSMA expression in the prostate of TRAMP mice (**A**) Expression levels of *DPP4* in the prostate. Left: Protein levels of DPP4 determined by Western blot. β-Actin was used as a loading control. Representative blots from two animals per group are shown. Densitometric values (mean ± SD), normalized to the STD group, are displayed. Right: *DPP4* mRNA expression assessed by qPCR and normalized to *GAPDH.* (**B**) Quantification of *PSMA* and *AR* mRNA levels in prostate tissue from TRAMP mice fed with STD or STD + CAP, as determined by qPCR. *GAPDH* was used as the housekeeping gene. Data are presented as mean ± SD. *P<*0.05 (*), *P<*0.01 (**) and *P<*0.001 (***) indicate significant differences by unpaired *t*-test.

These findings also raise the possibility that CAP may counteract the down-regulation of DPP4, a gene proposed to function as an AR-regulated tumor suppressor. To explore this mechanism further, we measured DPP4 levels in prostate tissue at the end of the experiment using Western blot. As shown in Figure 7A, left panel, CAP supplementation led to a significant increase in DPP4 expression. To confirm this finding, we analyzed DPP4 mRNA levels using qPCR, which also showed an increase in the CAP-fed group (Figure 7A, right panel). The down-regulation of DPP4 in castration-resistant PC has been linked to AR activity [11]. To assess whether CAP also restored AR activity, we determined the expression of the AR and of the PSMA,t time that capsaicin, a bioactive compo a well-established AR-regulated gene. CAP administration in animal diets, while not affecting AR levels (Figure 7B, left panel), induced an increase in PSMA mRNA expression (Figure 7B, right panel), suggesting that CAP may restore AR activity.

These results indicate that the *in vivo* treatment with CAP can restore the expression of DPP4 that decreases during the progression of PC.

## Discussion

The present study provides compelling evidence that DPP4 plays a tumor-suppressive role in PC and that its expression may be up-regulated by CAP. Consistent with previous reports showing that DPP4 expression declines during the transition to CRPC [11,12,15], we observed reduced DPP4 levels in androgen-resistant LN-Flu cells relative to androgen-sensitive LNCaP cells. Notably, this down-regulation paralleled diminished AR expression and decreased levels of canonical AR target genes, PSA and PSMA, reinforcing the notion that DPP4 is an AR-regulated gene whose expression is compromised in CRPC.

Importantly, we demonstrate for the first time that CAP, a bioactive compound with known antiproliferative properties [31–35], can restore DPP4 expression both *in vitro* and *in vivo*. Although CAP treatment led to a paradoxical reduction in AR and AR target genes *in vitro*, it significantly up-regulated DPP4 in both LNCaP and LN-Flu cells, suggesting an AR-independent mechanism. This observation is supported by our *in vivo* TRAMP model data, where dietary CAP restored DPP4 expression, increased PSMA mRNA levels, and suppressed key markers of cancer stemness (Nanog and Oct4). Furthermore, CAP activated the AMPK signaling pathway, consistent with previous findings in prostate cells [36,38]. As AMPK activation has been linked to tumor suppression and metabolic reprogramming, its role in mediating DPP4 restoration warrants further investigation.

Our data strongly support a functional role for DPP4 in modulating PC cell behavior. Lentiviral overexpression of DPP4 led to up-regulation of AR and PSMA in both LNCaP and LN-Flu cells, further supporting its contribution to maintaining AR signaling. These results align with Russo et al., who identified DPP4 as an AR-stimulated tumor suppressor gene [11]. Mechanistically, we found that DPP4 overexpression suppressed EMT, as evidenced by proteomic studies and Western blot analysis showing increased E-cadherin and decreased N-cadherin expression. Conversely, DPP4 overexpression reduced the expression of the metastasis-associated glycoprotein EpCAM, whereas DPP4 knockdown increased its expression. The down-regulation of EpCAM, a marker frequently associated with therapy resistance in PC [40], suggests that DPP4 may mitigate the invasive and proliferative potential of tumor cells. Functionally, DPP4 overexpression led to significant reductions in cell migration, invasion, and proliferation, as demonstrated by wound healing and CFSE-based assays. These phenotypic changes are consistent with prior studies reporting anti-migratory and anti-invasive roles of DPP4 in other cancer contexts [14,15]. In melanoma, the expression of DPP4 is lost in melanocytes during the later stages of malignant transformation, and similar to our results, re-expression of DPP4, either via an inducible tetracycline-regulated vector or through ectopic expression using cDNA transfection, led to a marked reduction in invasiveness, tumorigenicity, and overall oncogenic phenotype, shifting the cells toward a more normal melanocyte-like state [41]. Intriguingly, DPP4 not only exerted these effects independently, but also enhanced the antiproliferative action of CAP, highlighting a possible synergistic relationship that could be therapeutically exploited.

Current epidemiological evidence does not support a clear association between the clinical use of DPP4 inhibitors and the incidence of PC. Interestingly, a retrospective analysis of the SEER-Medicare database reported improved overall survival among diabetic patients with PC receiving DPP4 inhibitors, suggesting a potential beneficial association in the clinical setting, although the observational nature of the study precludes causal inference [10]. Likewise, long-term use of sitagliptin may reduce PC risk in male patients with type 2 diabetes—potentially by up to 35% compared with nonusers. This reduced incidence was particularly notable in men who used the medication continuously for more than 12 months [8,9]. While gliptins may help lower the initial risk of developing PC, their effect on existing tumors is more complex. Importantly, these epidemiological findings should not be directly compared with experimental studies involving DPP4 depletion or genetic loss of DPP4 expression. Pharmacological DPP4 inhibitors, such as sitagliptin, selectively inhibit the catalytic activity of the enzyme while preserving DPP4 protein expression and its non-enzymatic functions, including interactions involved in immune regulation and cell signaling. In contrast, genetic depletion or down-regulation eliminates both enzymatic and structural functions of DPP4, potentially leading to broader biological consequences. Indeed, experimental studies have demonstrated that DPP4 down-regulation can accelerate progression to castration-resistant PC, supporting a tumor-suppressive role for DPP4 expression under androgen-deprived conditions, whereas pharmacological inhibition may produce more nuanced and context-dependent effects. Moreover, its transgenic overexpression led to apoptosis, cell cycle arrest, and the inhibition of *in vitro* cell migration and invasion [41]. In support of these findings, other studies concluded that DPP4 is an epigenetically regulated tumor suppressor in castration-resistant PC [14]. Therefore, discrepancies between clinical epidemiological data and mechanistic studies likely reflect the fundamental biological differences between transient enzymatic inhibition and complete loss of DPP4 function, emphasizing that conclusions derived from genetic depletion models should not be directly extrapolated to patients treated with DPP4 inhibitors.

Taken together, our findings underscore the multifaceted role of DPP4 in PC biology. While previous epidemiological studies suggested a protective effect of DPP4 inhibitors in type 2 diabetic patients [9,42], our data, in agreement with emerging evidence [14–16], support a tumor-suppressive role for DPP4 expression in androgen-resistant PC, particularly in the context of AR signaling and disease progression to CRPC. These contrasting findings highlight the complexity of DPP4 biology, which involves both enzymatic and non-enzymatic mechanisms. That is, the DPP4 proteolytic activity seems to mediate tumor responses that are distinct from those mediated by its chaperone function, and the balance between them is influenced by tissue context, disease stage, and hormonal milieu, with the protein interaction activity becoming dominant in the androgen-resistant stage.

Our results provide evidence of a unique role for DPP4 chaperone activity in androgen resistance, leading to inhibition of cell migration, which may contribute to an emerging anticancer strategy for improved antitumor efficacy in precision medicine.

## Conclusion

Our study identifies DPP4 as a key mediator of PC suppression, acting through restoration of AR signaling, inhibition of EMT, and suppression of tumor cell proliferation and migration. CAP emerges as a novel agent capable of up-regulating DPP4 expression and counteracting features of castration resistance. These findings not only provide mechanistic insight into the role of DPP4 in PC but also open new avenues for therapeutic intervention in CRPC. Further studies are needed to determine the clinical applicability of targeting the DPP4 axis, particularly in combination with metabolic modulators such as CAP.

## Supplementary Material

Supplementary Figures S1-S6

## Data Availability

The datasets generated and/or analyzed during the present study are available in the ZENODO repository, 10.5281/zenodo.17045739. All R scripts, analysis templates, and raw/processed data files are available in the GitHub repository linked to the present study: https://github.com/Belen-G-Sanchez/Proteomic-Data-Associated-with-DPP4-Overexpression-.git.

## Abbreviations

ACC: acetyl-CoA carboxylase
ADT: androgen deprivation therapy
AMPK: AMP-activated kinase
AR: androgen receptor
ATCC: American Type Culture Collection
CAP: capsaicin
CFSE: carboxyfluorescein succinimidyl ester
CRPC: castration-resistant prostate cancer
DIA: data-independent acquisition
DEPs: differentially expressed proteins
DPP4: dipeptidyl peptidase-4
Dox: doxycycline
EpCAM: epithelial cell adhesion molecule
EMT: epithelial-to-mesenchymal transition
FDR: false discovery rate
FBS: fetal bovine serum
PC: prostate cancer
PSA: prostate-specific antigen
PSMA: prostate-specific membrane antigen
qPCR: quantitative real-time PCR
SD: standard deviation
STD: standard diet

## References

[1] Siegel R.L., Kratzer T.B., Giaquinto A.N., Sung H. and Jemal A. (2025) Cancer statistics, 2025. CA Cancer J. Clin. 75, 10–45 10.3322/caac.21871 39817679 PMC11745215

[2] Filho A.M., Laversanne M., Ferlay J., Colombet M., Pineros M., Znaor A. et al. (2024) The GLOBOCAN 2022 cancer estimates: data sources, methods, and a snapshot of the cancer burden worldwide. Int. J. Cancer 156, 1336–1346 10.1002/ijc.35278 39688499

[3] Paralkar D., Akbari A. and Aron M. (2024) Prostatic adenocarcinoma: molecular underpinnings and treatment-related options. Urol. Oncol. 42, 203–210 10.1016/j.urolonc.2024.03.003 38508940

[4] Dunaevsky Y.E., Tereshchenkova V.F., Oppert B., Belozersky M.A., Filippova I.Y. and Elpidina E.N. (2020) Human proline specific peptidases: a comprehensive analysis. Biochim. Biophys. Acta Gen. Subj. 1864, 129636 10.1016/j.bbagen.2020.129636 32433934

[5] Sebastian-Martin A., Sanchez B.G., Mora-Rodriguez J.M., Bort A. and Diaz-Laviada I. (2022) Role of dipeptidyl peptidase-4 (DPP4) on COVID-19 physiopathology. Biomedicines 10, 2026 10.3390/biomedicines10082026 36009573 PMC9406088

[6] Pacheco R., Martinez-Navio J.M., Lejeune M., Climent N., Oliva H., Gatell J.M. et al. (2005) CD26, adenosine deaminase, and adenosine receptors mediate costimulatory signals in the immunological synapse. Proc. Natl. Acad. Sci. U.S.A. 102, 9583–9588 10.1073/pnas.0501050102 15983379 PMC1172240

[7] Havre P.A., Abe M., Urasaki Y., Ohnuma K., Morimoto C. and Dang N.H. (2008) The role of CD26/dipeptidyl peptidase IV in cancer. Front. Biosci. 13, 1634–1645 10.2741/2787 17981655

[8] Lin Y., Xu G., Li L., Xiang J. and Zhai L. (2024) Incretin-based drugs decrease the incidence of prostate cancer in type 2 diabetics: a pooling-up analysis. Medicine (Baltimore). 103, e38018 10.1097/MD.0000000000038018 38758855 PMC11098233

[9] Lu S., Yin H., Yu O.H.Y. and Azoulay L. (2022) Incretin-based drugs and the incidence of prostate cancer among patients with type 2 diabetes. Epidemiology 33, 563–571 10.1097/EDE.0000000000001486 35394977

[10] Shah C., Hong Y.R., Bishnoi R., Ali A., Skelton W.P.t., Dang L.H. et al. (2020) Impact of DPP4 inhibitors in survival of patients with prostate, pancreas, and breast cancer. Front. Oncol. 10, 405 10.3389/fonc.2020.00405 32296640 PMC7136489

[11] Russo J.W., Gao C., Bhasin S.S., Voznesensky O.S., Calagua C., Arai S. et al. (2018) Downregulation of dipeptidyl peptidase 4 accelerates progression to castration-resistant prostate cancer. Cancer Res. 78, 6354–6362 10.1158/0008-5472.CAN-18-0687 30242112 PMC6239953

[12] Li Q.K., Chen J., Hu Y., Hoti N., Lih T.M., Thomas S.N. et al. (2021) Proteomic characterization of primary and metastatic prostate cancer reveals reduced proteinase activity in aggressive tumors. Sci. Rep. 11, 18936 10.1038/s41598-021-98410-0 34556748 PMC8460832

[13] Nazarian A., Lawlor K., Yi S.S., Philip J., Ghosh M., Yaneva M. et al. (2014) Inhibition of circulating dipeptidyl peptidase 4 activity in patients with metastatic prostate cancer. Mol. Cell. Proteomics 13, 3082–3096 10.1074/mcp.M114.038836 25056937 PMC4223493

[14] Wesley U.V., McGroarty M. and Homoyouni A. (2005) Dipeptidyl peptidase inhibits malignant phenotype of prostate cancer cells by blocking basic fibroblast growth factor signaling pathway. Cancer Res. 65, 1325–1334 10.1158/0008-5472.CAN-04-1852 15735018

[15] Wen Y.C., Lin C.Y., Hsiao C.H., Wang S.S., Huang H.C., Lin Y.W. et al. (2023) Genetic variants of dipeptidyl peptidase IV are linked to the clinicopathologic development of prostate cancer. J. Cell. Mol. Med. 27, 2507–2516 10.1111/jcmm.17845 37533175 PMC10468658

[16] Sabater A., Sanchis P., Seniuk R., Pascual G., Anselmino N., Alonso D.F. et al. (2024) Unmasking neuroendocrine prostate cancer with a machine learning-driven seven-gene stemness signature that predicts progression. Int. J. Mol. Sci. 25, 11356 10.3390/ijms252111356 39518911 PMC11545501

[17] Metri N.A., Mandl A. and Paller C.J. (2025) Harnessing nature’s therapeutic potential: a review of natural products in prostate cancer management. Urol. Oncol. 43, 221–243 10.1016/j.urolonc.2024.12.260 39794185

[18] Sanchez A.M., Sanchez M.G., Malagarie-Cazenave S., Olea N. and Diaz-Laviada I. (2006) Induction of apoptosis in prostate tumor PC-3 cells and inhibition of xenograft prostate tumor growth by the vanilloid capsaicin. Apoptosis 11, 89–99 10.1007/s10495-005-3275-z 16374544

[19] Mori A., Lehmann S., O'Kelly J., Kumagai T., Desmond J.C., Pervan M. et al. (2006) Capsaicin, a component of red peppers, inhibits the growth of androgen-independent, p53 mutant prostate cancer cells. Cancer Res. 66, 3222–3229 10.1158/0008-5472.CAN-05-0087 16540674

[20] Stewart S.A., Dykxhoorn D.M., Palliser D., Mizuno H., Yu E.Y., An D.S. et al. (2003) Lentivirus-delivered stable gene silencing by RNAi in primary cells. RNA 9, 493–501 10.1261/rna.2192803 12649500 PMC1370415

[21] Waterborg J.H. and Matthews H.R. (1984) The lowry method for protein quantitation. In Proteins Methods in Molecular Biology™(Walker J., ed.), p. 1, Humana Press 10.1385/0-89603-062-8:1 20512668

[22] Karousi P., Voumvouraki M., Nikolaou P.E., Kollias I., Paradeisi F., Sampanai E. et al. (2025) Easy Proteomics Sample Preparation: Technical Repeatability and Workflow Optimization Across 8 Biological Matrices in a New Core Facility Setting. Proteomics 25, 15–24 10.1002/pmic.70064 41132037 PMC12576925

[23] Kong A.T., Leprevost F.V., Avtonomov D.M., Mellacheruvu D. and Nesvizhskii A.I. (2017) MSFragger: ultrafast and comprehensive peptide identification in mass spectrometry-based proteomics. Nat. Methods 14, 513–520 10.1038/nmeth.4256 28394336 PMC5409104

[24] Yu F., Haynes S.E., Teo G.C., Avtonomov D.M., Polasky D.A. and Nesvizhskii A.I. (2020) Fast quantitative analysis of timsTOF PASEF data with MSFragger and IonQuant. Mol. Cell. Proteomics 19, 1575–1585 10.1074/mcp.TIR120.002048 32616513 PMC7996969

[25] Demichev V., Messner C.B., Vernardis S.I., Lilley K.S. and Ralser M. (2020) DIA-NN: neural networks and interference correction enable deep proteome coverage in high throughput. Nat. Methods 17, 41–44 10.1038/s41592-019-0638-x 31768060 PMC6949130

[26] Cox J., Hein M.Y., Luber C.A., Paron I., Nagaraj N. and Mann M. (2014) Accurate proteome-wide label-free quantification by delayed normalization and maximal peptide ratio extraction, termed MaxLFQ. Mol. Cell. Proteomics 13, 2513–2526 10.1074/mcp.M113.031591 24942700 PMC4159666

[27] Wu T., Hu E., Xu S., Chen M., Guo P., Dai Z. et al. (2021) clusterProfiler 4.0: a universal enrichment tool for interpreting omics data. Innovation (Camb) 2, 100141 10.1016/j.xinn.2021.100141 34557778 PMC8454663

[28] Yu G. (2024) Thirteen years of clusterProfiler. Innovation (Camb) 5, 100722 10.1016/j.xinn.2024.100722 39529960 PMC11551487

[29] Yu G., Wang L.G. and He Q.Y. (2015) ChIPseeker: an R/Bioconductor package for ChIP peak annotation, comparison and visualization. Bioinformatics 31, 2382–2383 10.1093/bioinformatics/btv145 25765347

[30] Ni J., Cozzi P., Hao J., Beretov J., Chang L., Duan W. et al. (2013) Epithelial cell adhesion molecule (EpCAM) is associated with prostate cancer metastasis and chemo/radioresistance via the PI3K/Akt/mTOR signaling pathway. Int. J. Biochem. Cell Biol. 45, 2736–2748 10.1016/j.biocel.2013.09.008 24076216

[31] Sanchez A.M., Malagarie-Cazenave S., Olea N., Vara D., Chiloeches A. and Diaz-Laviada I. (2007) Apoptosis induced by capsaicin in prostate PC-3 cells involves ceramide accumulation, neutral sphingomyelinase, and JNK activation. Apoptosis 12, 2013–2024 10.1007/s10495-007-0119-z 17828457

[32] Sanchez B.G., Bort A., Mateos-Gomez P.A., Rodriguez-Henche N. and Diaz-Laviada I. (2019) Combination of the natural product capsaicin and docetaxel synergistically kills human prostate cancer cells through the metabolic regulator AMP-activated kinase. Cancer Cell Int. 19, 54 10.1186/s12935-019-0769-2 30899201 PMC6408806

[33] Zhu M., Yu X., Zheng Z., Huang J., Yang X. and Shi H. (2020) Capsaicin suppressed activity of prostate cancer stem cells by inhibition of Wnt/beta-catenin pathway. Phytother. Res. 34, 817–824 10.1002/ptr.6563 31782192

[34] Ramos-Torres A., Bort A., Morell C., Rodriguez-Henche N. and Diaz-Laviada I. (2016) The pepper's natural ingredient capsaicin induces autophagy blockage in prostate cancer cells. Oncotarget 7, 1569–1583 10.18632/oncotarget.6415 26625315 PMC4811481

[35] Malagarie-Cazenave S., Olea-Herrero N., Vara D. and Diaz-Laviada I. (2009) Capsaicin, a component of red peppers, induces expression of androgen receptor via PI3K and MAPK pathways in prostate LNCaP cells. FEBS Lett. 583, 141–147 10.1016/j.febslet.2008.11.038 19059400

[36] Sanchez B.G., Bort A., Mora-Rodriguez J.M. and Diaz-Laviada I. (2022) The natural chemotherapeutic capsaicin activates AMPK through LKB1 kinase and TRPV1 receptors in prostate cancer cells. Pharmaceutics 14, 329 10.3390/pharmaceutics14020329 35214061 PMC8880011

[37] Hurwitz A.A., Foster B.A., Allison J.P., Greenberg N.M. and Kwon E.D. (2001) The TRAMP mouse as a model for prostate cancer. Curr. Protoc. Immunol., Chapter 20 45, 20.5.1–20.5.23 10.1002/0471142735.im2005s45 18432778

[38] Lee M.S., Doo M., Kim I.H. and Kim Y. (2024) Effects of capsicum oleoresin on the energy expenditure and mitochondrial content of brown adipose tissue in mice fed a high-fat diet. Prev. Nutr. Food Sci. 29, 422–429 10.3746/pnf.2024.29.4.422 39759824 PMC11699576

[39] Chen Z., Liu J., Ding H., Yan C., Zhu H., Huang S. et al. (2024) Dietary supplementation with capsaicinoids alleviates obesity in mice fed a high-fat-high-fructose diet. Food Funct. 15, 8572–8585 10.1039/D4FO02102A 39073607

[40] Liao Y., Wu M., Jia Y., Mou R. and Li X. (2022) EpCAM as a novel biomarker for survivals in prostate cancer patients. Front. Cell Dev. Biol. 10, 843604 10.3389/fcell.2022.843604 35517503 PMC9065552

[41] Wesley U.V., Albino A.P., Tiwari S. and Houghton A.N. (1999) A role for dipeptidyl peptidase IV in suppressing the malignant phenotype of melanocytic cells. J. Exp. Med. 190, 311–322 10.1084/jem.190.3.311 10430620 PMC2195594

[42] Chou O.H.I., Lu L., Chung C.T., Chan J.S.K., Chan R.N.C., Lee A.Y.H. et al. (2025) Comparisons of the risks of new-onset prostate cancer in type 2 diabetes mellitus between SGLT2I and DPP4I users: a population-based cohort study. Diab. Metab. 51, 101571 10.1016/j.diabet.2024.101571 39182669

