# Supplementary material for: Proteomic profiling reveals that DPP4 overexpression increases cell adhesion, inhibits cell migration, and restores androgen sensitivity in prostate cancer
Source: Biosci Rep. 2026 Sep 4;46(9):BSR20260054. doi: 10.1042/BSR20260054 (PMC13542909; doi:10.1042/BSR20260054)
Supplement: Supplementary file 1 [file BSR-2026-0054_supp.pdf]

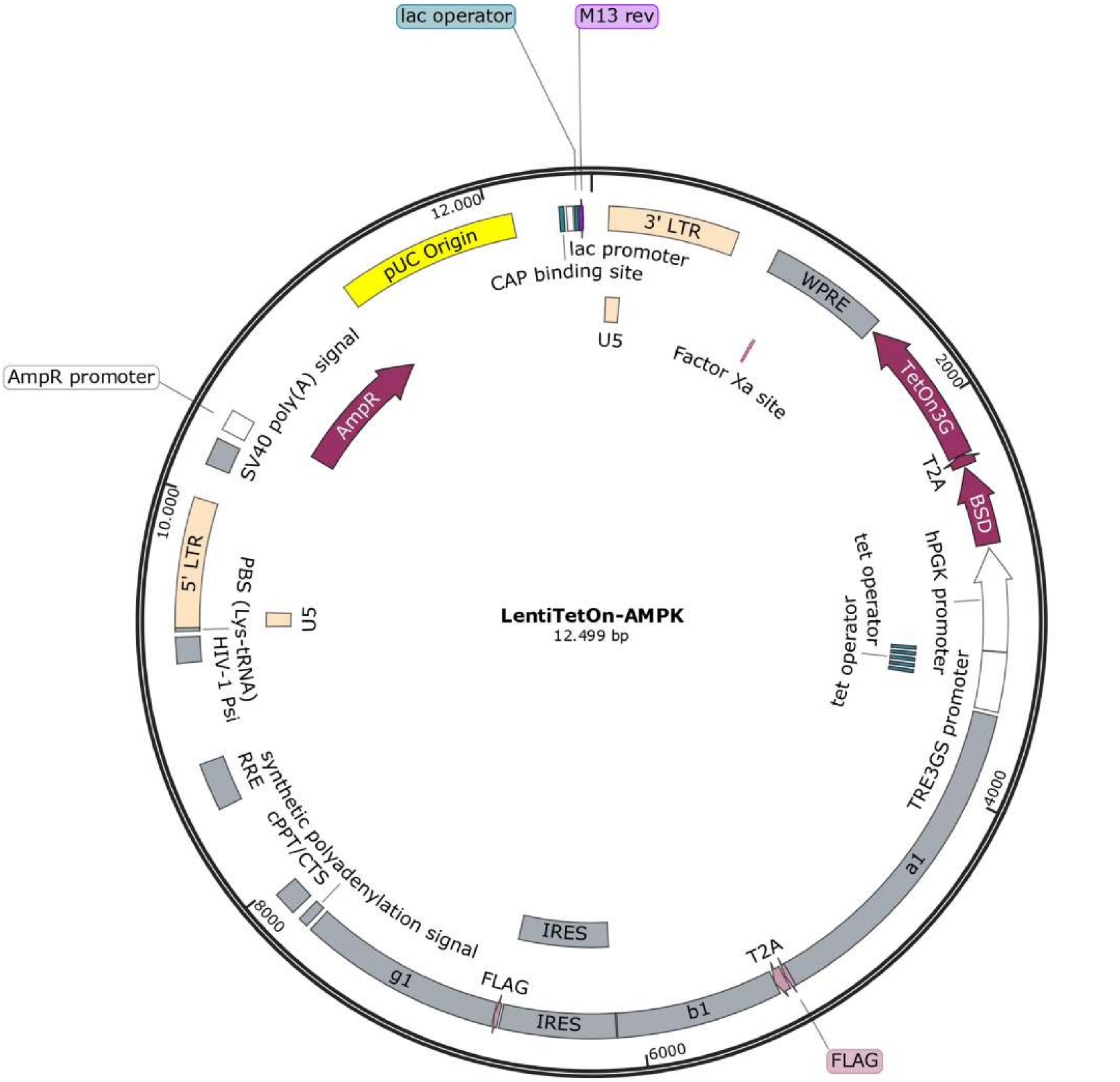

Supplementary Figure 1. Schematic map of the doxycycline-inducible LentiTetON–AMPK expression vector constructed in-house.

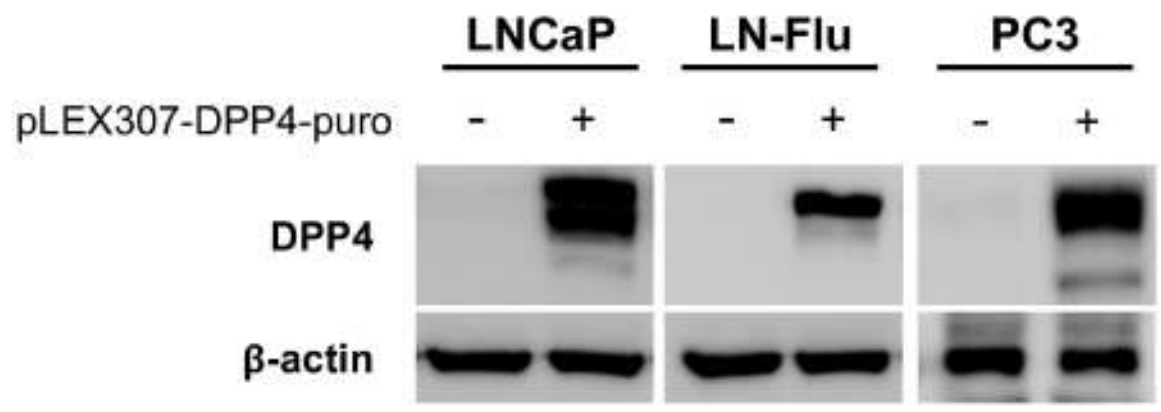

Supplementary Figure 2. DPP4 levels in DPP4-infected LNCaP, LN-Flu, and PC3 cells were determined by Western blot.

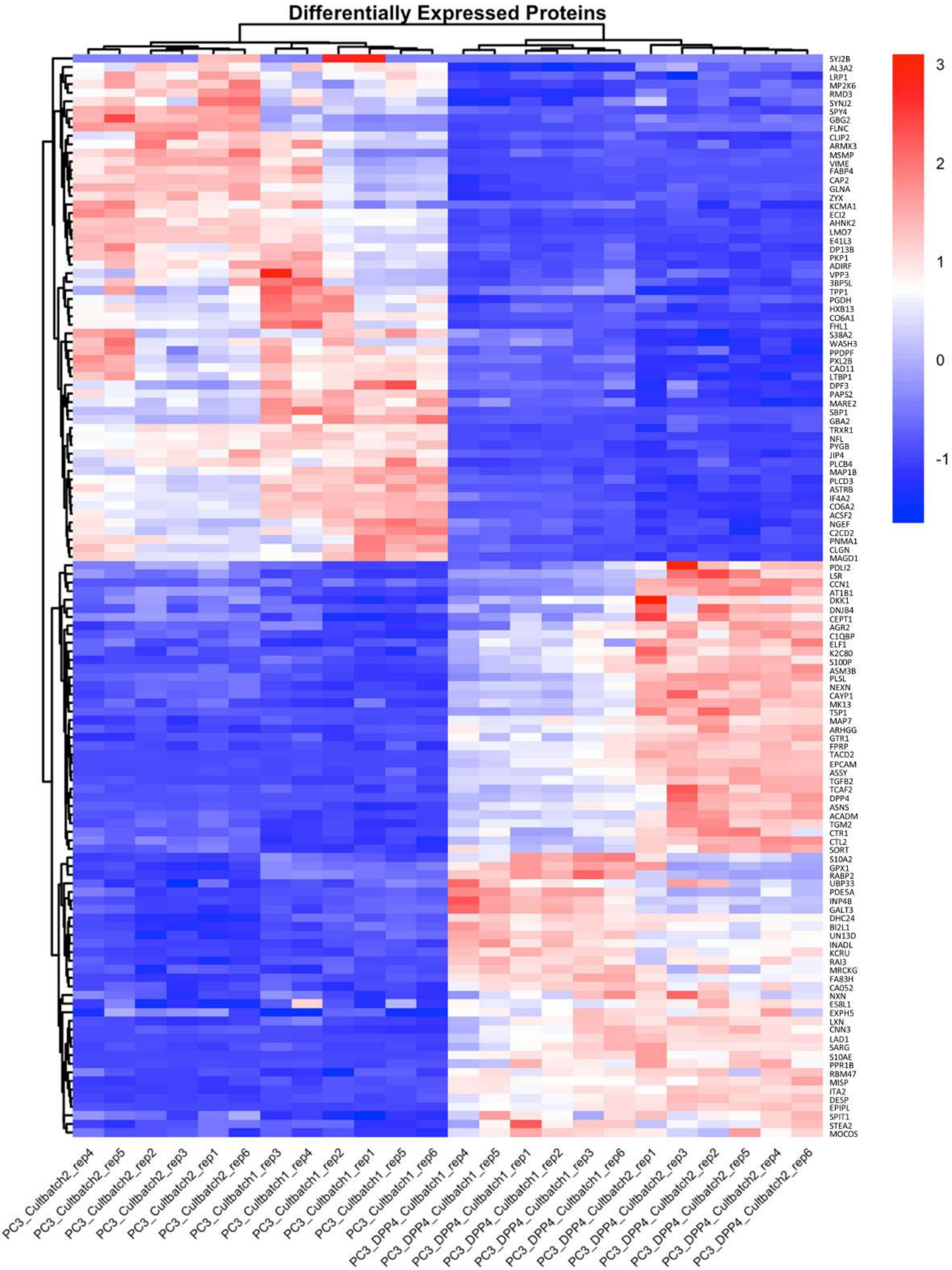

Supplementary Figure 3. Expanded heatmap of differentially expressed proteins between wild-type and DPP4-overexpressing PC3 cells. Magnified view of Figure 4B showing hierarchical clustering of samples and DEPs ($\log_2$ fold change ≥ |1.2|) between PC3 wild-type and DPP4-overexpressing cells.

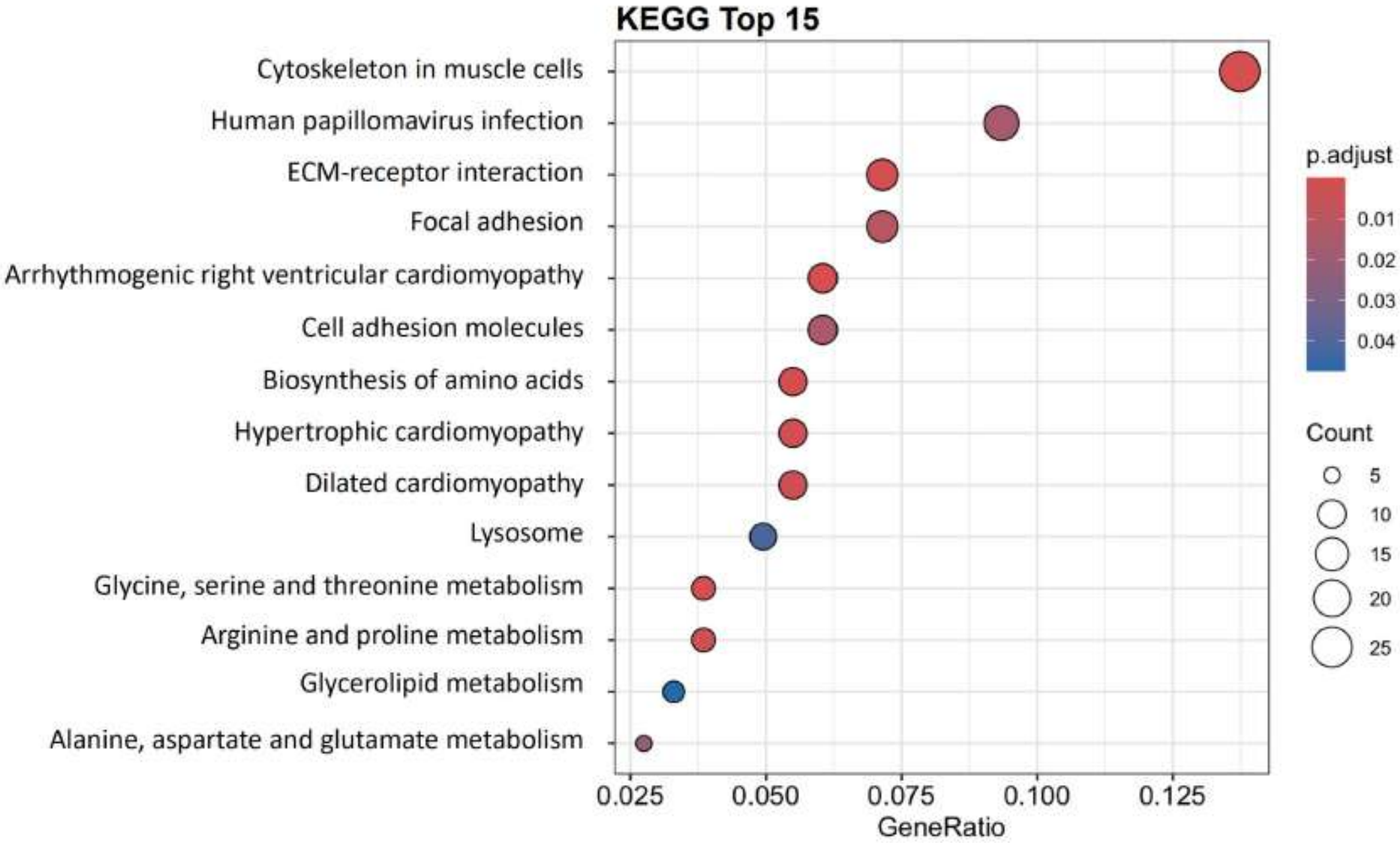

Supplementary Figure 4. Complementary analyses of the proteomic assay. KEGG (Kyoto Encyclopedia of Genes and Genomes) pathway enrichment analysis of differentially expressed proteins (Log_2 fold change > |1|, $p < 0.05$ comparing PC3-WT and PC3-DPP4 cells.

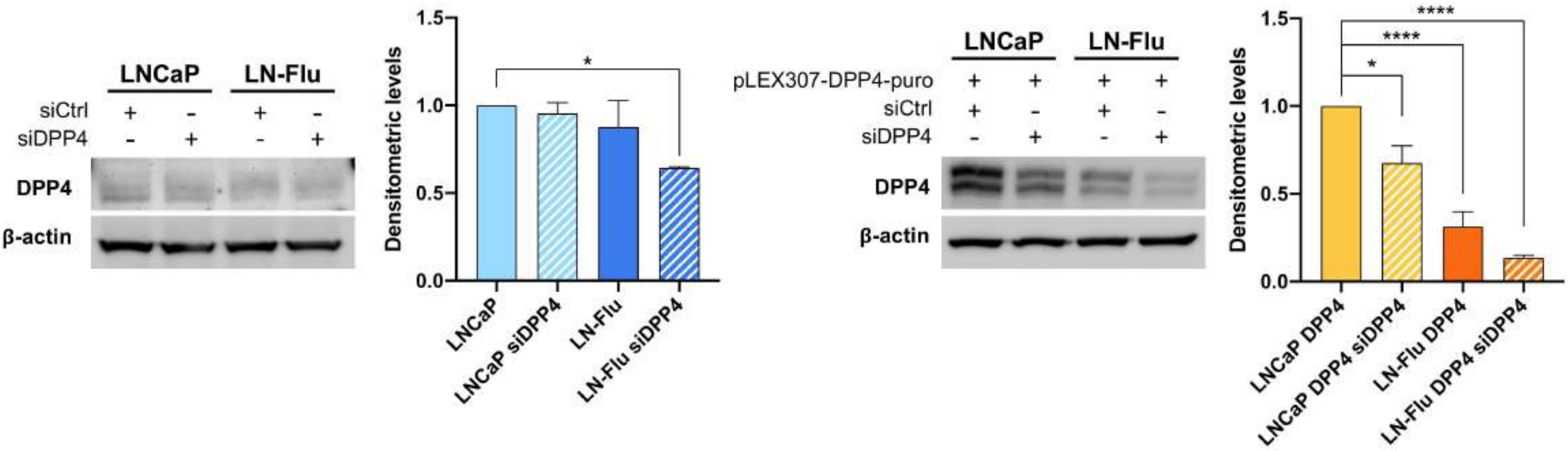

Supplementary Figure 5. DPP4 protein expression following DPP4 silencing in LNCaP and LN-Flu cells. Representative Western blots showing DPP4 protein expression in LNCaP and LN-Flu cells transfected with either control siRNA (siCtrl) or DPP4-specific siRNA (siDPP4) are shown on the left. The right panels show representative Western blots of LNCaP and LN-Flu cells stably overexpressing DPP4 and subsequently transfected with either siCtrl or siDPP4. β-Actin was used as the loading control. Data are presented as the mean ± SD (n = 3). Statistical significance was determined by two-way ANOVA followed by Tukey's multiple comparisons test. $p < 0.05$ (*), $p < 0.01$ (**), $p < 0.001$ (***), and $p < 0.0001$ (****).

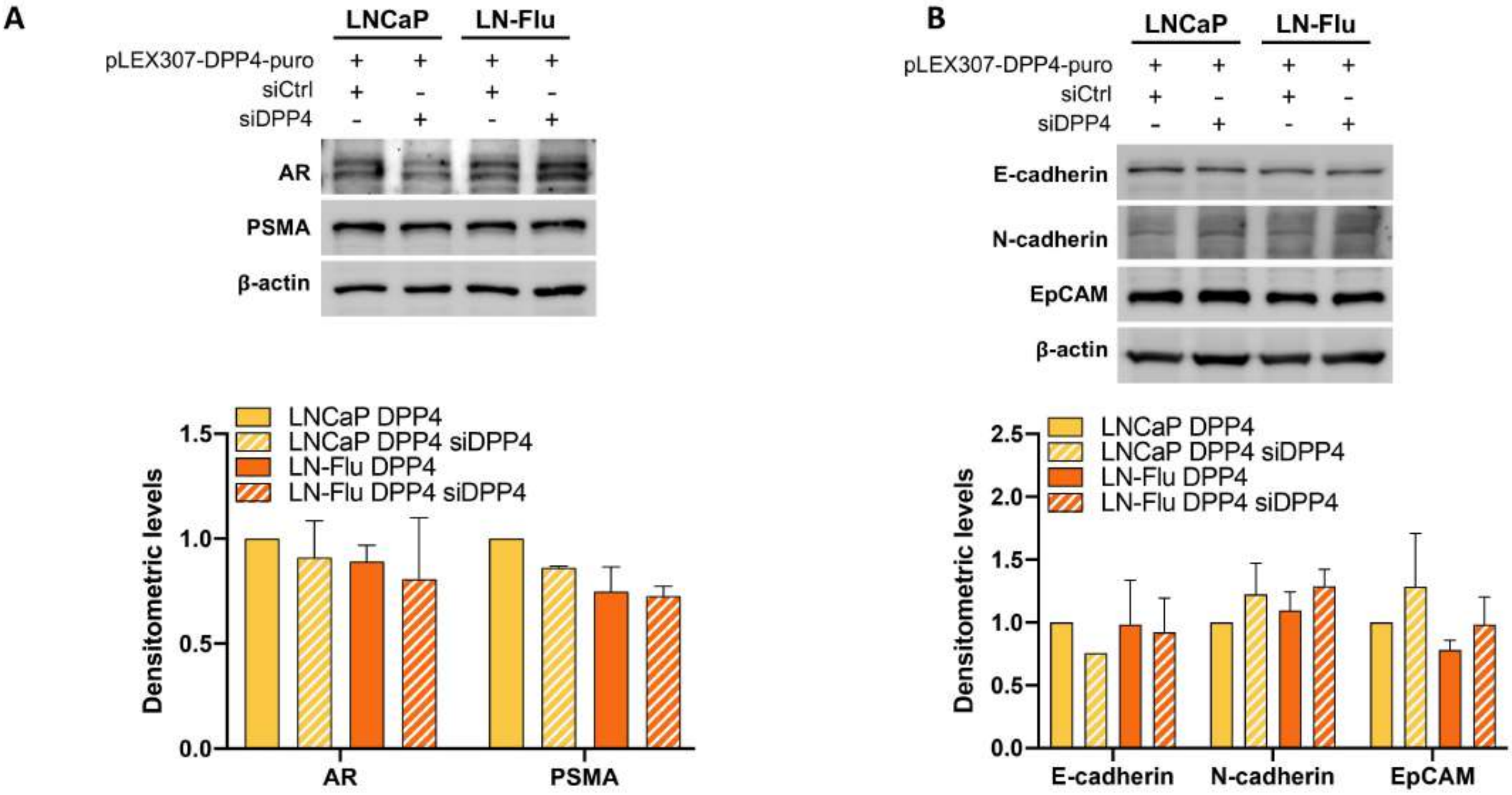

Supplementary Figure 6. Effects of DPP4 silencing in androgen-sensitive and androgen-insensitive prostate cancer cells. (A) Western blot analysis of AR and PSMA expression and **(B)** E-cadherin, N-cadherin, and EpCAM expression following DPP4 silencing in LNCaP and LN-Flu cells stably overexpressing DPP4. Representative immunoblots and the corresponding densitometric analyses are shown for each protein. β-Actin was used as the loading control. Data are presented as the mean ± SD (n = 3). Statistical significance was determined by two-way ANOVA followed by Tukey's multiple comparisons test. $p < 0.05$ (*), $p < 0.01$ (**), $p < 0.001$ (***), and $p < 0.0001$ (****).
